# Constructing cell-type-specific gene regulatory networks from cell–cell communication and a global gene regulatory network

**DOI:** 10.1093/bib/bbag525

**Published:** 2026-09-26

**Authors:** Yuke Xie, Bowen Fu, Laijun Zhong, Lei Wu, Haorong Li, Xiao Chang, Xiaoping Liu

**Affiliations:** Key Laboratory of Systems Health Science of Zhejiang Province, School of Life Science, Hangzhou Institute for Advanced Study, University of Chinese Academy of Sciences, No. 1 Xiangshan Branch Road, Xihu District, Hangzhou 310024, Zhejiang, China; Key Laboratory of Systems Health Science of Zhejiang Province, School of Life Science, Hangzhou Institute for Advanced Study, University of Chinese Academy of Sciences, No. 1 Xiangshan Branch Road, Xihu District, Hangzhou 310024, Zhejiang, China; Key Laboratory of Systems Health Science of Zhejiang Province, School of Life Science, Hangzhou Institute for Advanced Study, University of Chinese Academy of Sciences, No. 1 Xiangshan Branch Road, Xihu District, Hangzhou 310024, Zhejiang, China; Key Laboratory of Systems Health Science of Zhejiang Province, School of Life Science, Hangzhou Institute for Advanced Study, University of Chinese Academy of Sciences, No. 1 Xiangshan Branch Road, Xihu District, Hangzhou 310024, Zhejiang, China; School of Molecular Medicine, Hangzhou Institute for Advanced Study, University of Chinese Academy of Sciences, No. 1 Xiangshan Branch Road, Xihu District, Hangzhou 310024, Zhejiang, China; Institute of Statistics and Applied Mathematics, Anhui University of Finance and Economics, No. 962 Caoshan Road, Longzihu District, Bengbu 233030, Anhui, China; Key Laboratory of Systems Health Science of Zhejiang Province, School of Life Science, Hangzhou Institute for Advanced Study, University of Chinese Academy of Sciences, No. 1 Xiangshan Branch Road, Xihu District, Hangzhou 310024, Zhejiang, China

**Keywords:** single-cell RNA sequencing, cell-type-specific gene regulatory network, matrix decomposition

## Abstract

Accurately inferring cell-type-specific gene regulatory networks (GRNs) is crucial for understanding cellular heterogeneity, lineage determination, and disease progression mechanisms. Although single-cell RNA sequencing (scRNA-seq) enables high-resolution expression profiling, its inherent sparsity and high noise levels pose challenges to reliable GRN reconstruction. To address these issues, we propose cell type-specific gene regulatory network (CTN), a regulatory inference framework based on bilateral self-representation matrix decomposition, which integrates bulk RNA-seq data with scRNA-seq data to decompose global regulatory relationships and intercellular communication, thereby reconstructing GRNs at the cell type level. CTN demonstrates high inference accuracy and strong performance in functional module identification. In the analysis of real biological processes, CTN reveals the key regulators and functional modules in the differentiation process for different lineages of the hematopoietic system, identifies differentially perturbed regulatory networks for tumor-related cell types, and depicts potential functional mechanisms in the tumor microenvironment.

## Introduction

Gene regulatory networks (GRNs) play a crucial role in regulating gene expression programs that control cell function, behavior, and responses to environmental stimuli. Accurately inferring GRNs from transcriptomic data is a core task in systems biology and helps recover the regulatory mechanisms that drive complex biological processes. Although traditional GRN inference methods based on bulk RNA sequencing (RNA-seq) data have made significant progress, their analytical capabilities are limited to the average effect of cell populations at a tissue or organ level, making it difficult to reveal cell-type-specific regulatory signals in the tissue microenvironment. With the development of single-cell RNA sequencing (scRNA-seq), it has become possible to obtain gene expression profiles at single-cell resolution, laying the foundation for constructing cell-type-specific GRNs. Inferring cell-type-specific GRNs is important for elucidating how different cell types coordinate gene expression, identifying lineage-specific regulators, and understanding perturbation responses in cell differentiation or disease states, and is of great significance to developmental biology, regenerative medicine, and precision oncology. However, the sparsity and high noise of scRNA-seq data severely limit the direct inference of reliable GRNs. In recent years, some deep learning-based methods have been proposed to construct GRNs from scRNA-seq data, such as GRGNN [1], DeepDRIM [2], and DeepRIG [3]. However, most of the methods generally rely on prior knowledge of transcription factor (TF)-target pairs, which limits their applicability in unknown or scarce annotation systems.

In this study, we propose a new method to construct cell-type-specific GRNs, called cell type-specific gene regulatory network (CTN). This method is based on matrix decomposition and integrates bulk RNA-seq and scRNA-seq data with cell type labels. It can infer gene regulatory relationships in the same cell type without any prior information. CTN deconstructs the scRNA-seq expression matrix under the constraint of a bulk RNA-seq-derived global GRN to infer intercellular communication components, and further extracts cell-type-specific communication submatrices. It then reconstructs the expression matrix for the specific cell type to obtain cell-type-specific GRNs. In benchmark evaluations, CTN outperformed five classic GRN inference methods and showed higher inference accuracy and biological interpretability. In the analysis of real datasets, CTN successfully identifies key regulators and functional pathways that regulate cell differentiation and disease progression. This method not only effectively alleviates the impact of sparsity and noise of scRNA-seq data, but also deeply reveals the potential regulatory heterogeneity among cell types, providing a powerful tool for the extraction of functional-specific subnetworks and the analysis of potential regulatory mechanisms.

## Results

### Overview of CTN

**Figure 1:**
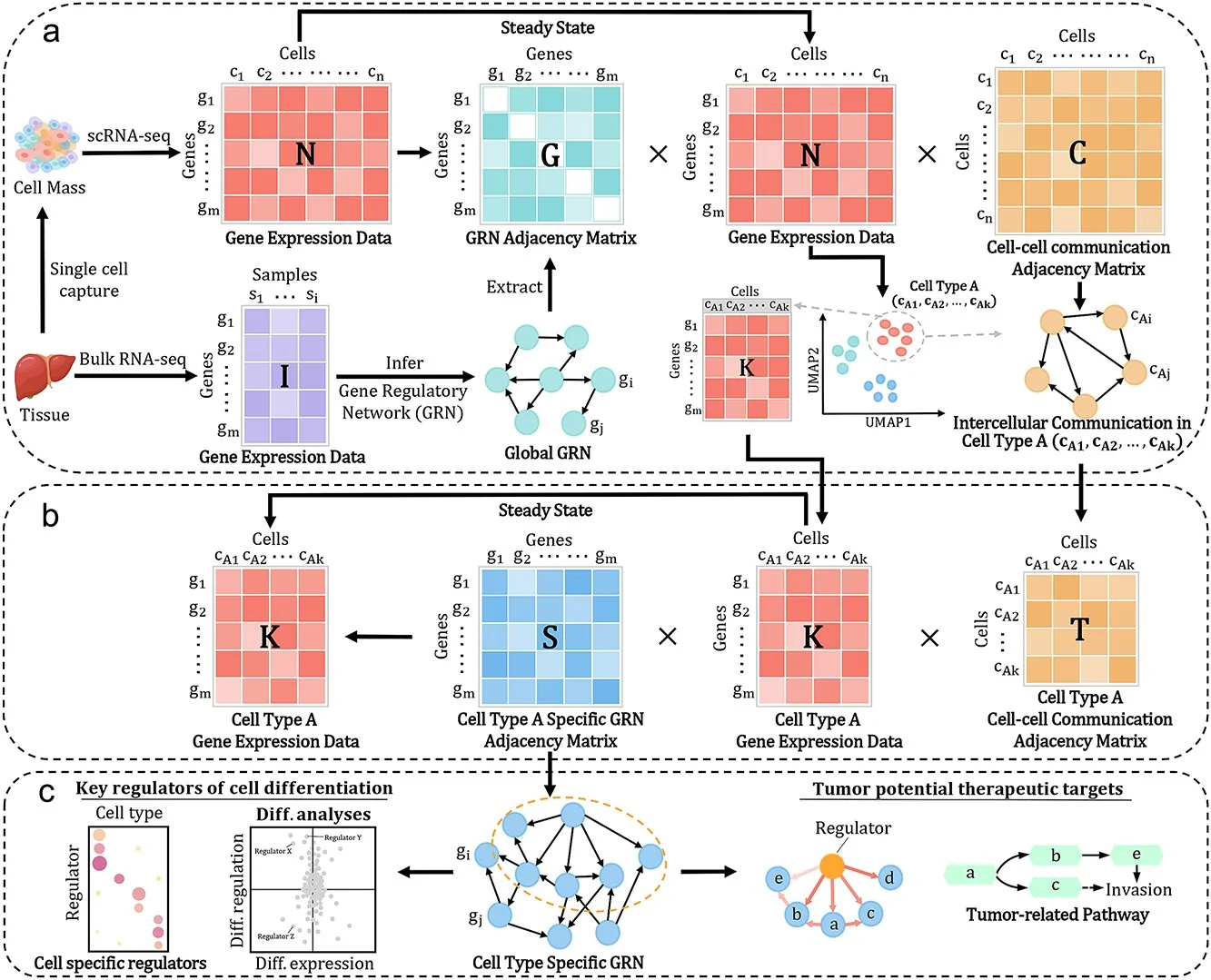
Overview of CTN; the CTN method is a bilateral self-representation model; (a) the method first self-decomposes the single-cell gene expression matrix to extract the gene regulatory relationships and cell–cell communication relationships that maintain homeostasis; the global GRN is constructed by bulk RNA-seq data from the same tissue, and the cell–cell communication matrix is obtained by solving the pseudo-inverse; (b) taking cell type A $\left({c}_{A1},{c}_{A2},\cdots, {c}_{Ak}\right)$ as an example, the cell–cell communication submatrix within the cell type is extracted, and the gene expression data belonging to cell type A is extracted from the original single-cell gene expression profile; by reconstructing the gene expression profile of cell type A, the pseudo-inverse is solved to obtain the final cell-type-specific GRN; (c) based on the constructed cell-type-specific GRN, subnetworks are extracted and applied to the study of cell differentiation and disease development, from which potential key regulators and regulatory mechanisms are identified.

CTN is designed to infer the specific GRN for each cell type from scRNA-seq data and bulk RNA-seq data of the same tissue, and effectively extract specific regulatory subnetworks that affect cell function. Meanwhile, CTN is also a data-driven algorithm to infer GRN without any prior knowledge, such as TF-target pairs. The maintenance of homeostasis of a tissue depends on the synergy of intrinsic gene regulation and extrinsic intercellular communication. The core of CTN is to adopt a two-step inference strategy: first, infer the cellular crosstalk relationship between cells by employing a matrix decomposition method [4, 5] (Fig. 1a). Second, classify and organize the intercellular communication in each cell type, and infer the cell-type-specific gene regulatory relationships through reverse reconstruction (Fig. 1b).

CTN is based on the idea of matrix decomposition and separates the gene and cell information from the gene expression matrix. Bulk RNA-seq data with *m* genes $\left({g}_1,{g}_2,\cdots, {g}_m\right)$ and *i* samples $\left({s}_1,{s}_2,\cdots, {s}_i\right)$ can be regarded as the matrix *I* of size *m× i*. The global GRN can be inferred for the *m* genes using matrix *I* and an adjacency matrix of size *m× m* can be subsequently obtained from the global GRN (Fig. 1a). Since the steady state of gene expression may result from the combined effects of gene regulation and intercellular communication, single-cell gene expression data with *m* genes $\left({g}_1,{g}_2,\cdots, {g}_m\right)$ and *n* cells $\left({c}_1,{c}_2,\cdots, {c}_n\right)$ can be regarded as a matrix *N* of size *m× n*, and the matrix *N* can be decomposed into three submatrices: the left submatrix *G* containing global gene regulation information, the original gene expression matrix *N*, and the right submatrix *C* containing cell–cell communication information (Equation 4 and Fig. 1a). The left submatrix *G* is the adjacency matrix of the global GRN from the matrix *I* (Fig. 1a). The right submatrix *C* of size *n× n* can be obtained by solving the pseudo-inverse matrix (Equation 6), which reflects the directed communication relationship among cells (Fig. 1a).

We then separate and organize the single-cell gene expression data of different cell types according to the cell type labels of *n* cells in the original single-cell expression data. We assume that *k* cells belong to cell type A $\left({c}_{A1},{c}_{A2},\cdots, {c}_{Ak}\right)$. The gene expression of cell type A is regarded as the matrix *K* of size *m× k* separated from matrix *N*. The matrix *K* can also be decomposed into three submatrices: the left submatrix *S* containing cell-type-specific gene regulatory information, the original gene expression matrix *K* of cell type A, and the right submatrix *T* containing the intercellular communication in cell type A (Equation 7 and Fig. 1b). The right submatrix *T* can be directly extracted from the matrix *C*. We can obtain the left submatrix *S* by solving the pseudo-inverse matrix (Equation 8 and Fig. 1b), which represents the directed regulatory relationship between *m* genes in cell type A, thereby constructing the cell-type-specific GRN (Fig. 1b).

By extracting cell-type-specific GRNs, we can identify key regulators that influence cell differentiation and disease progression. In this study, regulators are defined as genes that exhibit regulatory connections directed toward one or more downstream target genes (i.e. outgoing edges) within the extracted cell-type-specific GRNs. During cell differentiation, identifying key regulators in specific cell types can reveal cell-type-specific regulatory patterns and uncover mechanisms that may be overlooked by conventional gene expression analysis (Fig. 1c). During disease development, each regulator forms a subnetwork centered on itself by controlling the expression of its target genes, thereby elucidating tumor-related pathways underlying disease progression, and providing insights for precision medicine and personalized therapy (Fig. 1c).

### Performance on benchmark datasets

We systematically evaluated CTN using three benchmark datasets from the BEELINE [6] framework, a widely recognized benchmark for assessing GRN inference accuracy, together with bulk RNA-seq datasets from the corresponding tissues. The three datasets (Supplementary Note S9) mainly included human embryonic stem cells (hESC), human mature hepatocytes (hHEP), and mouse embryonic stem cells (mESC).

**Figure 2:**
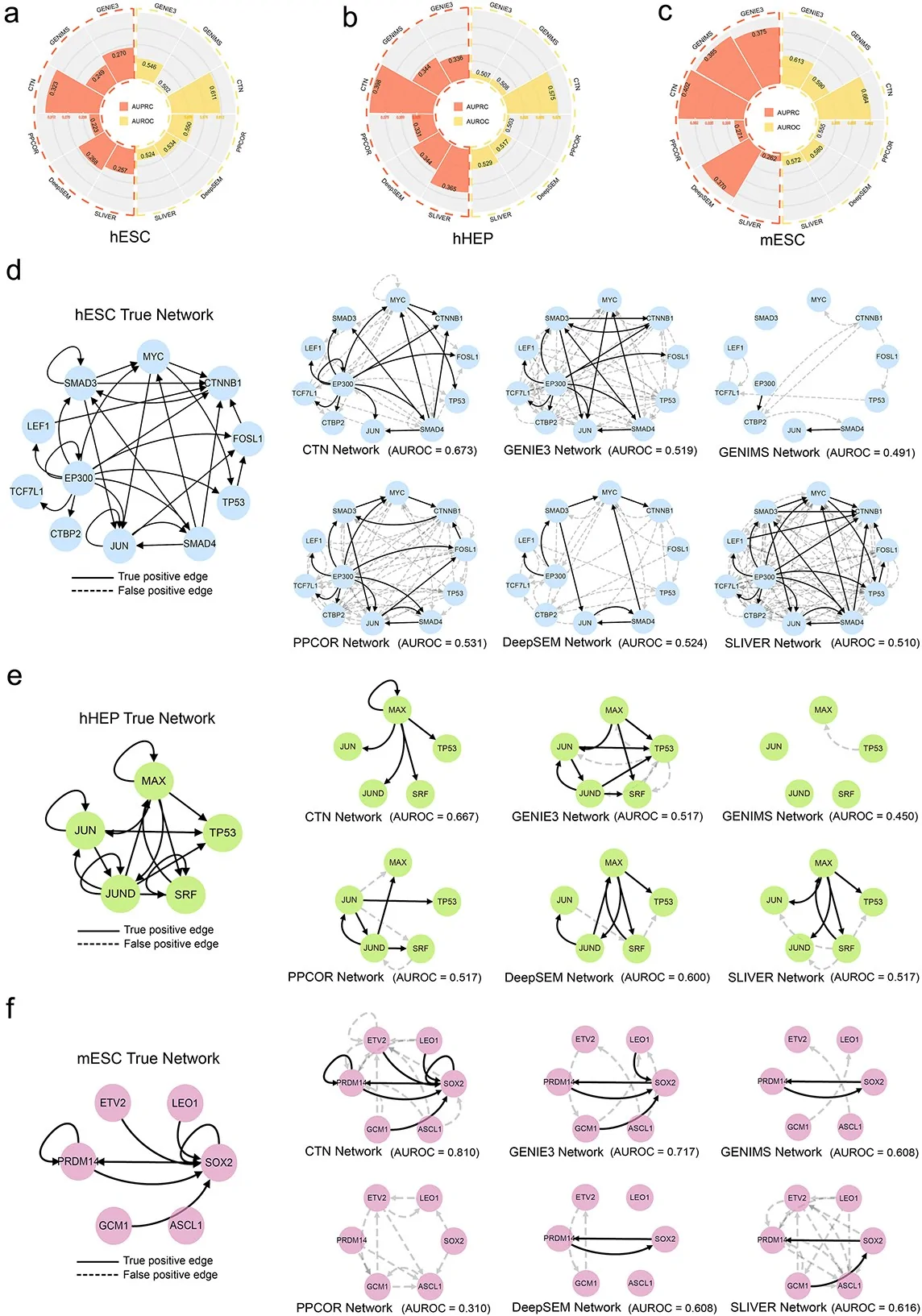
Performance comparison of CTN and other methods on three benchmark datasets; based on AUROC and AUPRC indicators, the CTN method performs best in overall prediction performance on (a) hESC, (b) hHEP, and (c) mESC datasets; in terms of evaluating the effectiveness of the method in extracting regulatory subnetworks with biological functions, the CTN method has a clear advantage in (d) hESC, (e) hHEP, and (f) mESC datasets.

To further quantitatively evaluate the accuracy of our method, we used the area under the receiver operating characteristic curve (AUROC) and area under the precision-recall curve metrics (Supplementary Note S9) to compare with five mainstream methods, including GENIE3 [7], GENIMS [8], PPCOR [9], DeepSEM [10], and SLIVER [11] (Supplementary Note S2). The results showed that CTN achieved the best performance in both metrics on the three datasets. In the AUROC metric, CTN outperformed the lowest method (GENIMS) by 10.9% on both the hESC (Fig. 2a and Supplementary Table S1) and mESC (Fig. 2c and Supplementary Table S1) datasets. It also outperformed the second-ranked method (SLIVER) by 4.6% on the hHEP dataset (Fig. 2b and Supplementary Table S1).

While accurately predicting the overall regulatory network structure, the CTN method also demonstrated strong performance in extracting function-specific subnetworks. In this study, function-specific subnetworks are defined as gene regulatory subnetworks composed of genes associated with particular biological functions. To evaluate the accuracy of identifying function-specific subnetworks, we performed Kyoto Encyclopedia of Genes and Genomes (KEGG; https://www.genome.jp/kegg/pathway.html) enrichment analysis on all genes included in cell-type-specific GRNs to identify pathways that play critical roles in the differentiation of the corresponding cell types, and extracted the function-specific subnetworks consisting of genes participating in differentiation-related pathways. Here, the true network is defined as the ground-truth GRN provided by the benchmark datasets, which represents the accepted and validated regulatory relationships among genes (Supplementary Note S10). Finally, we compared the accuracy of the predicted regulatory edges within function-specific subnetworks across different methods, where accuracy is defined as the AUROC value obtained by comparing the predicted regulatory edges against the corresponding true network from the benchmark dataset. For hESC, Wnt signaling can determine major developmental processes in the embryonic state and regulates maintenance, self-renewal, and differentiation of adult mammalian tissue stem cells [12]. Therefore, we focused on genes enriched in the Wnt signaling pathway and their regulatory relationships. The results showed that CTN achieved an average accuracy 15.8% higher than the other five methods (Fig. 2d). For hHEP, the mitogen-activated protein kinase (MAPK) signaling pathway supports the proliferation of hepatocytes *in vitro* and *in vivo* during hepatocyte differentiation [13]. We extracted the function-specific subnetworks composed of genes involved in the MAPK signaling pathway for comparative evaluation (Fig. 2e). CTN achieved an accuracy 6.7% higher than the second-ranked method (DeepSEM) (Fig. 2e). For mESC, the genes involved in the cell fate commitment biological process and their regulatory relationships were analyzed. Compared with other methods, CTN accurately predicted the main regulatory relationships among genes, with an AUROC value of 0.810 (Fig. 2f). These comparisons indicate that CTN can not only infer cell-type-specific GRNs, but also effectively extract specific subnetworks related to cell functions, thus providing a powerful tool for more detailed analysis of potential gene regulatory mechanisms affecting cell differentiation or disease development.

### Inferring cell-type-specific regulators in human hematopoiesis

**Figure 3:**
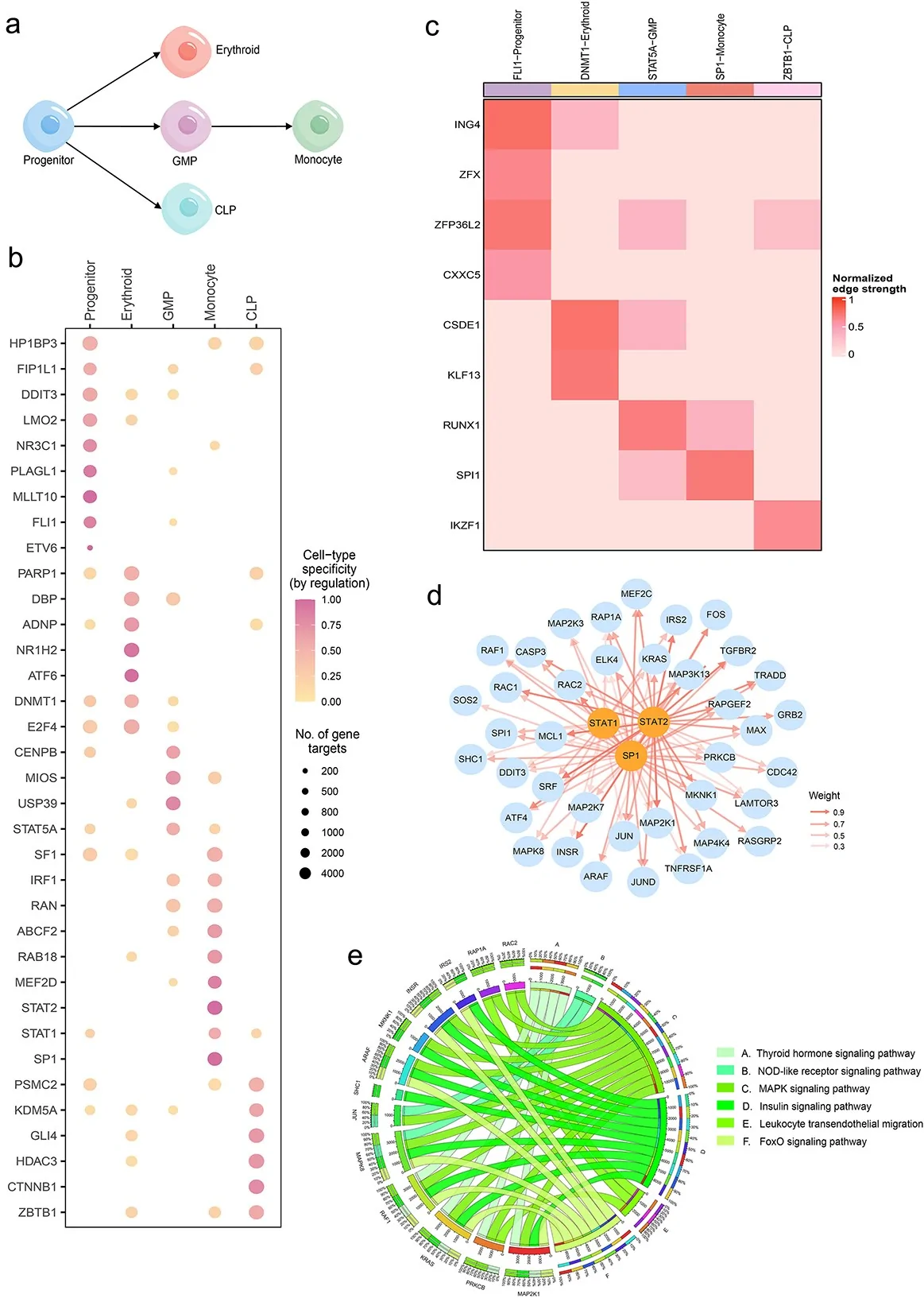
Analysis of cell-type-specific regulators in human hematopoietic differentiation dataset; (a) differentiation lineage diagram of some cells in the human hematopoietic process; (b) bubble diagram of regulators with significant specificity in each cell type; the size of the bubble indicates the number of target genes of the regulator, and the color of the bubble reflects the size of the specificity value; (c) highly regulated genes regulated by cell-type-specific regulators of each cell type; the rows in the figure represent cell-type-specific regulators of different cell types, and the columns represent target genes that are significantly regulated; (d) extract the cell-type-specific gene regulatory subnetwork of monocytes, and perform enrichment analysis on the genes contained in the network; (e) the chord diagram of enrichment analysis of monocytes shows the functional pathways in which genes are significantly enriched; the line connections in the chord diagram represent the association between each gene and different pathways.

To demonstrate the utility of the CTN method in the differentiation of various hematopoietic cell types, we collected a scRNA-seq dataset (Supplementary Table S8) containing bone marrow mononuclear cells from healthy donors [14] and a bulk RNA-seq dataset [15] from the same tissue. Based on the quantitative distribution of different cell types in the scRNA-seq data, we focused on five main cell types: hematopoietic stem cell (HSC), erythroid, granulocyte-monocyte progenitor (GMP), monocyte, and common lymphoid progenitor (CLP) (Fig. 3a), and employed CTN to construct GRNs for each cell type.

By comparing the GRNs of different cell types, CTN successfully identified a series of regulators exhibiting strong cell-type-specific regulatory weights (Supplementary Table S2). In this analysis, the regulatory weight refers to the post-processed edge weight in the cell-type-specific GRN, obtained after absolute-value transformation, normalization to the range of 0–1, and sparsity thresholding of the inferred regulatory coefficient. The average regulatory weight of a regulator is calculated as the mean of its outgoing regulatory edge weights to downstream target genes within a given cell-type-specific GRN, and is further used to calculate the cell-type-specific regulatory score as described in Supplementary Note S12.

As shown in Fig. 3b, we identified a total of 35 regulators across the five major cell types. These regulators displayed markedly higher average regulatory weights within their corresponding cell types compared to others, indicating distinct transcriptional control patterns. For example, ETV6 encodes a transcriptional repressor expressed in HSC (progenitor) and is involved in adult hematopoietic regulation [16]. E2F4 has been shown to act as an activating regulator during erythroid cell differentiation [17]. STAT5A is important for the proliferation and survival of the GMP lineage [18]. STAT1/2 plays a key role in the differentiation and maturation of monocytes [19, 20]. ZBTB1 is essential for T cell development and complete differentiation of B cells and natural killer (NK) cells in the CLP differentiation branch [21]. Notably, these cell-type-specific regulators did not show significant differences among different cell types in traditional gene expression analysis (Supplementary Fig. S2A), but exhibited distinct regulatory roles at the network level as revealed by CTN. Based on these cell-type-specific regulators, CTN further identified target genes with high regulatory weights (Fig. 3c). For example, in the HSC cell-type-specific network, FLI1 was predicted to regulate the target genes *ING4, ZFX*, and *CXXC5* (Fig. 3c). In HSCs, FLI1 is known as a key regulator that maintains the balance between HSC self-renewal and differentiation [22]. The target genes *ING4, ZFX*, and *CXXC5* are potentially involved in regulating the balance between HSC self-renewal and differentiation by mediating downstream pathways of FLI1 [23–25]. We also found that the predicted target genes of DNMT1 in erythroid cells were *CSDE1* and *KLF13* (Fig. 3c). In erythroid cells, DNMT1 has been reported to promote erythroid maturation and prevent apoptosis through stage-specific regulation [26]. The target gene *CSDE1* is required for the differentiation of erythroblasts into mature red blood cells [27], and *KLF13* has also been shown to be involved in the regulation of erythropoiesis [28]. In monocytes, SP1 was identified as a key regulator controlling the expression of SPI1 (Fig. 3c). SPI1 encodes an essential transcription factor that governs monocyte lineage commitment and differentiation [29]. SP1 has been reported to regulate monocyte-specific gene expression, including the activation of CD14, a membrane glycoprotein highly expressed on monocytes and macrophages [30].

To further explore the potential biological functions of the regulatory network centered on cell-type-specific regulators for cell differentiation in each cell type, we performed KEGG pathway enrichment analysis on genes regulated by cell-type-specific regulators in each cell type. In erythroid cells, the genes regulated by DNMT1 (Supplementary Fig. S2B) were mainly enriched in the thyroid hormone signaling pathway, mechanistic target of rapamycin (mTOR) signaling pathway, and forkhead box O (FoxO) signaling pathway (Supplementary Fig. S2C), all of which have important effects on the proliferation and differentiation of erythroid progenitor cells [31–33]. Among these pathways, STK11 and CSNK1E, which are regulated in the FoxO signaling pathway, activate FOXO3 through phosphorylation (Supplementary Fig. S2C). FOXO3 then has a certain acceleration effect on the differentiation of erythroid progenitor cells [34]. In monocytes, three major cell-type-specific regulators STAT1, STAT2, and SP1, along with their target genes *ARAF, RAF1*, and *JUN* (Fig. 3d), were mainly involved in the MAPK signaling pathway (Fig. 3e). MAPK signal transduction is an important mechanism for controlling basic processes such as growth, proliferation and migration, and hematopoietic differentiation may also be regulated by MAPK signal transduction [35]. In addition, monocyte differentiation may have also been found to be induced by the MAP3K–MKK–JNK pathway, with MKK denoting mitogen-activated protein kinase kinase and JNK denoting c-Jun N-terminal kinase [36]. In summary, CTN can not only discover key regulators that are difficult to identify by traditional expression analysis methods, but also further analyze downstream target genes and functional pathways for identifying specific regulatory subnetworks with clear cell function orientations and expanding our understanding of the regulatory mechanisms in the process of hematopoietic cell differentiation.

### Identifying lineage-specific regulators in human fetal hematopoietic differentiation

During cell differentiation, progenitor cells often contain multiple potential cell fates. Hematopoietic differentiation is a highly complex process in which different gene expression modules interact in a combinatorial manner to jointly regulate stemness maintenance, early lineage initiation, and final differentiation into the major branches of the hematopoietic system. The identification of lineage-specific regulators helps to reveal the fine molecular regulatory mechanisms and provide a theoretical basis for the deeper understanding of blood homeostasis, hematopoietic malignancies, and regenerative medicine [37, 38]. In this study, lineage-specific regulators are defined as genes that exert cell-type-specific regulatory effects at branch-point cell types, thereby influencing subsequent differentiation trajectories.

To examine the effectiveness of CTN in identifying lineage-specific regulators in those with known branching lineage structures, we apply CTN to public scRNA-seq [39] and bulk RNA-seq [40] datasets of human fetal hematopoiesis (Supplementary Table S9), where the single-cell datasets capture multiple blood lineage branching structures, including lymphoid-myeloid progenitor (LMP), granulocytic progenitor (GP), and MK-erythroid-mast progenitor (MEMP) branches (Supplementary Fig. S3A). We conduct a comprehensive analysis of the differential regulatory changes of regulators in cell-type-specific GRNs downstream of each branch of LMP, GP, and MEMP based on differential regulation (average regulatory weight) and differential expression. Lineage-specific regulators are characterized by differential regulation or non-differential expression and are likely to act as core determinants driving lineage commitment and specification.

**Figure 4:**
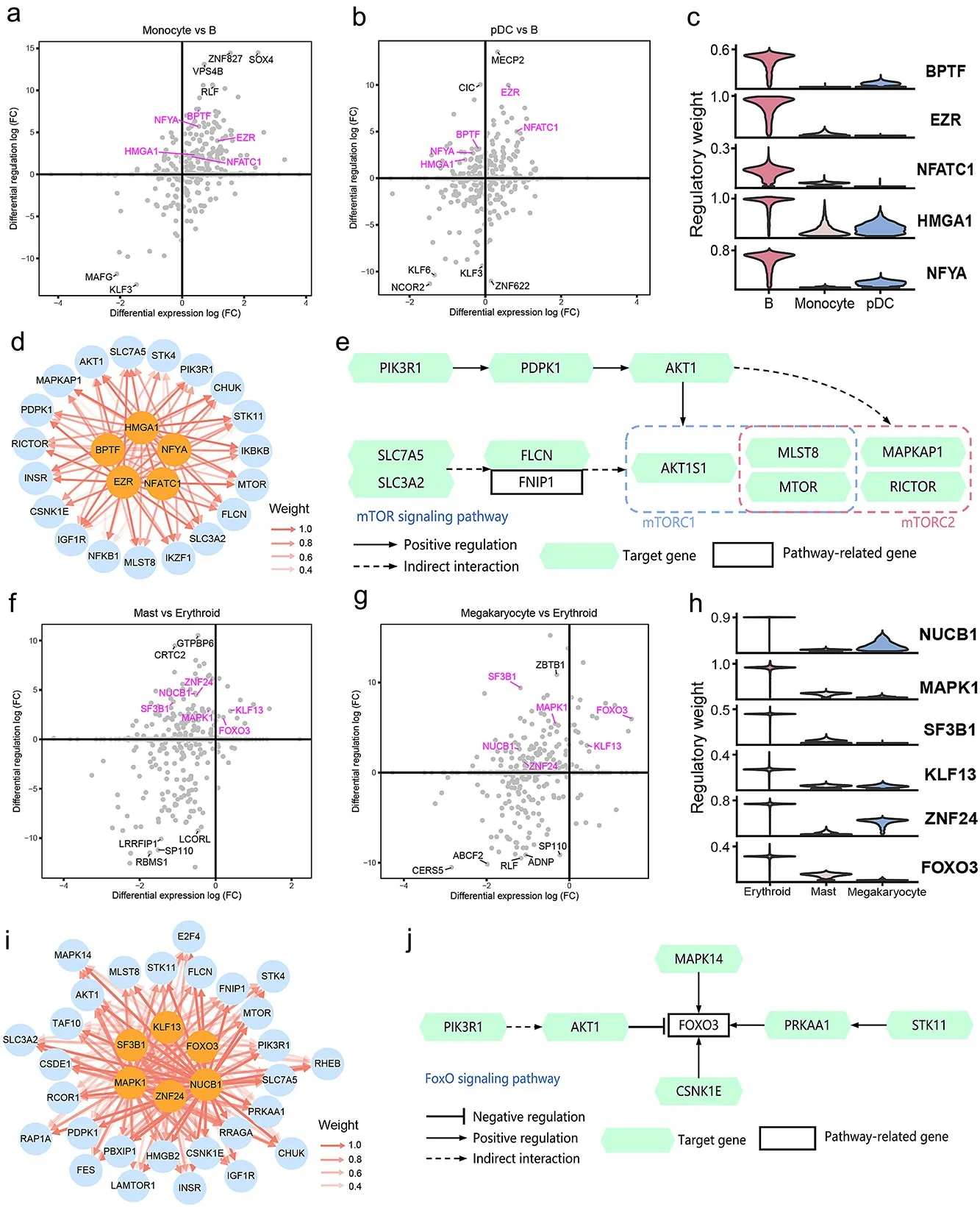
Analysis of lineage-specific regulators in human fetal hematopoietic differentiation data; in the LMP branch, compared B cells with monocytes (a) and B cells with pDCs (b) cell-type-specific GRNs, lineage-specific regulators that affect B cell differentiation are identified; (c) lineage-specific regulators have higher regulatory weights in B cells; (d) regulatory network composed of lineage-specific regulators and their target genes in B cells; (e) the main processes involved in the specific regulatory subnetwork of B cells in the mTOR signaling pathway; in the MEMP branch, comparing erythroid cells with mast cells (f) and erythroid cells with megakaryocytes (g) cell-type-specific GRNs, lineage-specific regulators that affect erythroid cell differentiation are identified; (h) lineage-specific regulators have higher regulatory weights in erythroid cells; (i) regulatory network composed of lineage-specific regulators and their target genes in erythroid cells; (j) the main processes involved in the FoxO signaling pathway by the specific regulatory subnetwork of erythroid cells.

In the LMP branch, we compared B cells with monocytes (Fig. 4a) and B cells with plasmacytoid dendritic cells (pDCs) (Fig. 4b) to identify lineage-specific regulators that exhibit distinct regulatory weight in B cells. We identified several candidate lineage-specific regulators that play specific regulatory roles in B cells: HMGA1, NFYA, BPTF, EZR, and NFATC1 (Supplementary Table S3). These lineage-specific regulators showed significantly higher log fold change values (Supplementary Note S13) in the differential regulation analysis, which can be observed in Fig. 4a and b, and have higher overall regulatory weights in B cells (Fig. 4c). Among lineage-specific regulators in B cells, HMGA1, through indirect interaction with DNA, acts as a transcriptional μ enhancer co-activator to regulate the μ enhancer required for B cell development [41]. BPTF and NFATC1 have been shown to play a key role in the normal differentiation and homeostasis of B cells [42, 43]. Furthermore, we extracted a regulatory subnetwork centered on lineage-specific regulators from the GRN of B cells (Fig. 4d), and performed KEGG pathway enrichment analysis on all genes contained in the subnetwork (Supplementary Fig. S3B). The genes were mainly enriched in the B cell receptor and mTOR signaling pathways (Supplementary Fig. S3B), which are essential for B cell survival and differentiation [44, 45]. Within the B cell subnetwork, CTN identified key genes enriched in the mTOR pathway, including PIK3R1, PDPK1, AKT1, mTOR, and RICTOR (Fig. 4e). The regulatory cascade revealed by CTN shows that PIK3R1 activates PDPK1, which phosphorylates AKT1 to activate mTORC1 and mTORC2 through the PI3K–AKT axis (Fig. 4e), and PDPK1 is essential for B cell survival and growth [46]. Additionally, SLC7A5, SLC3A2, and FLCN were identified as target genes within the B cells subnetwork (Fig. 4d). The SLC7A5–SLC3A2 and FLCN–FNIP1 complexes mediate amino acid transport and sensing, providing metabolic input for full mTORC1 activation (Fig. 4e) [47, 48]. Downstream, mTORC1 drives biosynthetic programs supporting B cell proliferation, whereas mTORC2 ensures B cell development [44, 49]. MAPKAP1 and RICTOR, identified as target genes within the B cells subnetwork (Fig. 4d), are core components of the mTORC2 complex (Fig. 4e) and play essential roles in regulating B cell metabolism and fate determination [50, 51]. In the MEMP branch, by comparing erythroid cells with mast cells (Fig. 4f) and erythroid cells with megakaryocytes (Fig. 4g), we identified several candidate lineage-specific regulators that play specific regulatory roles in erythroid cells: KLF13, FOXO3, NUCB1, MAPK1, ZNF24, and SF3B1 (Supplementary Table S4). Of particular note, decreased SF3B1 activity significantly affects erythroid progenitors and terminal differentiation [52]. KLF13 is involved in the normal control of erythroid cell generation [53]. FOXO3 plays a key role in erythroid differentiation and integrates different signals to regulate gene expression during erythroid cell generation [34]. In the downstream cell types of the MEMP branch, including erythroid cells, mast cells, and megakaryocytes (Supplementary Fig. S3A), the lineage-specific regulators have a higher overall regulatory weight in erythroid cells (Fig. 4h). To further understand the main functions of these lineage-specific regulators in erythroid differentiation, we performed functional pathway enrichment (Supplementary Fig. S3C) on the erythroid lineage-specific regulators subnetwork reconstructed by CTN (Fig. 4i). The FoxO signaling pathway was identified as a significantly enriched pathway (Supplementary Fig. S3C), known to play a crucial role in driving erythroid differentiation [33]. From the erythroid subnetwork (Fig. 4i), several target genes, including PIK3R1, AKT1, STK11, MAPK14, CSNK1E, and PRKAA1, are identified as key components associated with the FoxO signaling pathway. Therefore, we reconstructed the main regulatory processes of the FoxO signaling pathway based on these target genes (Fig. 4j). During erythropoiesis, PIK3R1 indirectly activates AKT1 to inhibit FOXO3 activity to maintain precursor cell proliferation (Fig. 4j) and enhance the cell response to erythropoietin; this process strictly controls the expansion, maturation, and survival of erythroid progenitor cells to ensure erythropoiesis [33]. MAPK14-, CSNK1E-, and STK11-mediated PRKAA1 restimulates FOXO3 activity (Fig. 4j) to initiate differentiation-related gene expression under oxidative stress and metabolic challenges, among which PRKAA1 is required for autophagy-dependent mitochondrial clearance during erythrocyte maturation [54].

Similarly, we also identified several potential lineage-specific regulators during the differentiation of two other cell types downstream of the LMP branch: monocytes (Supplementary Figure S4 and Supplementary Table S5) and pDCs (Supplementary Figure S5 and Supplementary Table S6), as detailed in Supplementary Note S3. Overall, the CTN method can effectively identify regulatory subnetworks with lineage-specific regulators as the core. These candidate lineage-specific regulators not only play a wide range of known roles in the hematopoietic system, but may also be involved in the biological processes of specific lineage determination.

We also considered the potential influence of cell-number imbalance in the fetal hematopoietic differentiation dataset. Cell numbers substantially varied across cell types, ranging from 1357 hematopoietic stem cell–multipotent progenitor (HSC-MPP) cells to 96 megakaryocytes (Supplementary Table S9). CTN is a matrix decomposition-based network inference framework rather than a supervised classification model and does not involve a classification loss function weighted by cell type abundance. In addition, CTN reconstructs cell-type-specific GRNs separately for each cell type and compares normalized regulatory weights rather than raw total edge weights, which helps reduce scale effects caused by differences in cell number and matrix size. Therefore, the inferred cross-cell-type regulatory patterns are less likely to be driven primarily by cell number differences. Nevertheless, results from low-abundance cell types, such as megakaryocytes, were interpreted cautiously, with emphasis on qualitative network topology and biological consistency rather than small quantitative differences in regulatory weight.

### Analysis of cell-type-specific gene regulatory networks in normal colorectal tissue

In order to explore the application value of CTN in revealing the potential regulatory mechanisms in the development of diseases, this study first conducts a systematic analysis of the specific regulatory networks of different cell types in the healthy group, aiming to identify key regulatory modules and potential core functional genes under steady-state conditions, thereby providing a reliable reference baseline for the comparison of network perturbations under subsequent disease states.

**Figure 5:**
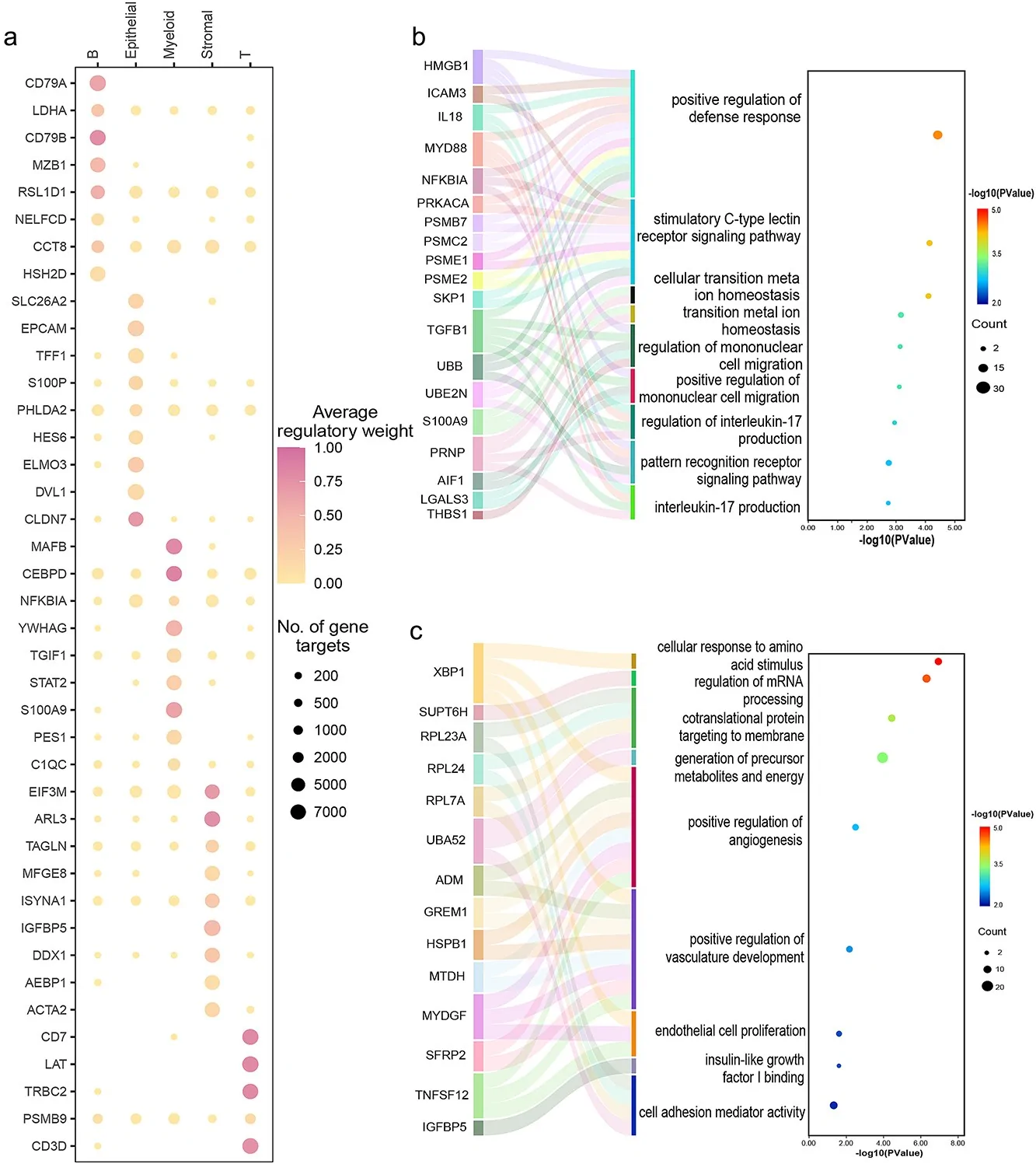
Analysis of cell-type-specific GRNs in normal colorectal tissue; (a) bubble diagram of regulators with significant specificity in each cell type; the size of the bubble indicates the number of target genes of the regulator, and the color of the bubble reflects the average regulatory weight; the biological processes involved in genes in various cell-type-specific GRNs are analyzed, including (b) myeloid cells and (C) stromal cells.

We used the human colorectal cancer (CRC) scRNA-seq [55] dataset (Supplementary Table S10), which covers cell populations from healthy adjacent tissues and tumor core areas, and integrated bulk RNA-seq data from The Cancer Genome Atlas (TCGA). The data were divided into healthy and diseased groups, and cell-type-specific GRNs were constructed using the expression profiles of each cell type in the healthy group. By comparing the GRNs of each cell type, we systematically identified regulators with significant regulatory weight in each cell type. The identified regulators were closely associated with cell identity and function across multiple cell types (Supplementary Note S4). These representative regulators were characterized by high cell-type-specific regulatory weight (Fig. 5a and Supplementary Note S4).

To further elucidate the biological functions of the identified regulators across different cell types, we performed KEGG enrichment analysis of the biological processes in which these regulators participate. The analysis was based on the regulators identified from each cell type, including the representative regulators shown in Fig. 5a, aiming to reveal potential functional roles in maintaining cellular homeostasis and specific activities under normal physiological conditions. The enrichment results (Fig. 5b and c and Supplementary Fig. S6) showed that the regulators identified from each cell type are significantly associated with diverse biological processes critical for tissue integrity and immune balance (Fig. 5b and c and Supplementary Fig. S6), including Fc-epsilon receptor signaling, responses to oxidative and endoplasmic reticulum stress, vasculature development, and regulation of immune effector processes. These enriched pathways underscore the essential roles of cell-type-specific regulators in maintaining the steady-state functionality of distinct cell populations within healthy colorectal tissue (Supplementary Note S5). Together, these findings demonstrate that the regulatory networks inferred by CTN are not only statistically cell type-specific but also biologically meaningful, as they converge on key molecular processes supporting the characteristic physiological functions of each cell type (Supplementary Note S5). Importantly, this confirms that CTN captures cell-type-specific regulatory architectures, thereby providing a robust network-level foundation for subsequent analyses of differential regulatory perturbations during disease onset and progression.

### Revealing the variation of cell-type-specific gene regulatory networks in colorectal cancer and healthy controls

To investigate CRC-associated changes in cell-type-specific regulatory networks, we constructed cell-type-specific GRNs for healthy and CRC samples and performed differential analysis of regulatory weights within the same cell type [56]. In this study, network perturbation refers to disease-associated changes in regulatory relationships within cell-type-specific GRNs, rather than changes in gene expression levels alone. Based on these differences, we constructed differential perturbation networks to identify perturbed regulators and their downstream target genes, aiming to reveal potential regulatory mechanisms involved in CRC development. In the tumor immune microenvironment, myeloid cells and stromal cells are widely believed to play key roles in tumor occurrence and development. Myeloid-derived suppressor cells (MDSCs) are types of heterogeneous myeloid cells that can suppress immune responses through multiple mechanisms and have an important impact on tumor immune escape [57–59]. In addition, cancer-associated fibroblasts (CAFs), as the most important stromal cell type in the tumor microenvironment, regulate epithelial-mesenchymal transition (EMT) by secreting extracellular matrix, cytokines, and growth factors, thereby promoting tumor growth, metastasis, and drug resistance [60, 61]. Therefore, we focused on changes in regulatory network weights in myeloid and stromal cells between healthy and CRC states to provide potential target references for anti-tumor immunotherapy.

**Figure 6:**
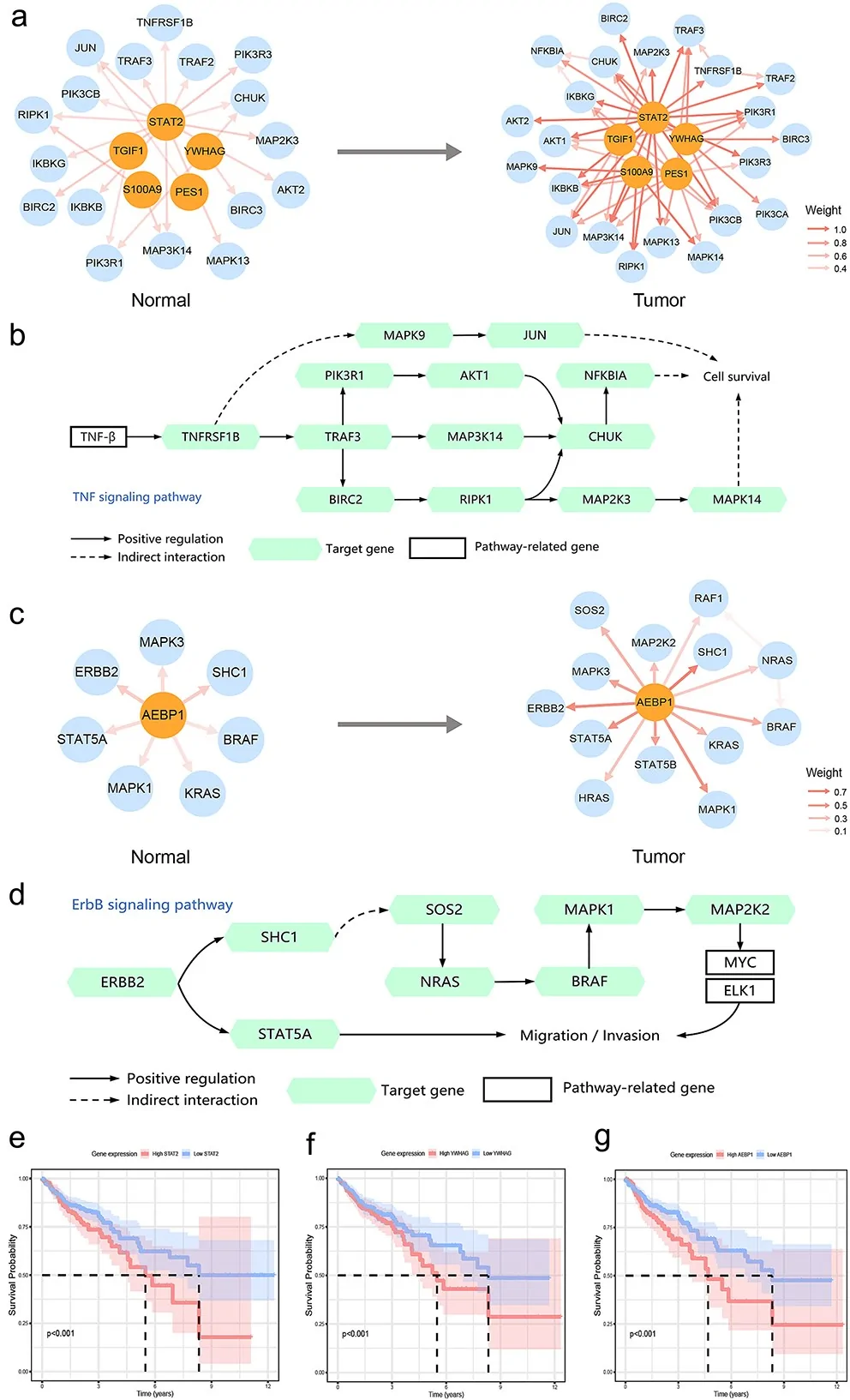
Uncovering the potential regulatory mechanisms in CRC; (a) differential changes in GRN between normal and disease groups of myeloid cells; (b) main branches of TNF signaling pathway involved in perturbation network genes in myeloid cells; (c) differential changes in GRN between normal and disease groups of stromal cells; (d) main branches of ErbB signaling pathway involved in perturbation network genes in stromal cells; prognostic survival analysis is performed on (e) STAT2, (f) YWHAG, and (g) AEBP1, and upregulation of gene expression is associated with poor prognosis.

In myeloid cells, regulators were ranked according to the magnitude of disease-associated changes in average regulatory weight, and regulators exhibiting both strong regulatory influence and marked network perturbation were prioritized as key regulators. STAT2, PES1, TGIF1, YWHAG, and S100A9 were identified as key regulators in myeloid cells (Fig. 6a), indicating substantial alterations in downstream regulatory programs during CRC progression. Among them, STAT2 can promote tumorigenesis by activating proinflammatory mediators [62]; downregulation of PES1 expression can significantly inhibit the proliferation of CRC cells [63]; TGIF1 and YWHAG act as tumor promoters and promote the migration and invasion of CRC cells [64, 65]. In addition, MDSCs enhance CRC cell stemness and growth via exosomal S100A9 [66]. To elucidate the biological consequences of regulatory changes, enrichment analysis was performed on target genes showing significant regulatory differences in disease states. The enrichment analysis revealed that these target genes were predominantly involved in CRC-related pathways (Supplementary Fig. S7A), with the tumor necrosis factor (TNF) signaling pathway emerging as a central regulatory axis (Fig. 6b). TNF signaling is known to regulate the survival, accumulation, and immunosuppressive function of MDSCs within the tumor microenvironment [67]. Consistent with this role, the perturbed regulatory network converged on tumor necrosis factor receptor 2 (TNFR2)-mediated signaling, accompanied by activation of downstream PI3K-AKT and noncanonical nuclear factor kappa B (NF-κB) pathways involving AKT1, MAPK14, and MAPK9 (Fig. 6b). These signaling cascades promote anti-apoptotic capacity, metabolic adaptation, and immunosuppressive activity of MDSCs, thereby facilitating immune evasion and tumor progression [68, 69]. In stromal cells, CTN analysis identified AEBP1 as a regulator exhibiting pronounced disease-specific changes in regulatory weight (Fig. 6c). AEBP1 was therefore selected as a representative key regulator in stromal cells for further analysis. AEBP1 is highly expressed in CAFs and can promote tumor development by inducing EMT [70]. We analyzed the differences in the status of the AEBP1 regulatory network in healthy controls and CRC (Fig. 6c) and performed KEGG enrichment analysis on the differential target genes of AEBP1 (Supplementary Fig. S7B). We selected the ErbB signaling pathway with significant enrichment of AEBP1 target genes for subsequent analysis, which is essential for regulating the EMT process (Fig. 6d). Activation of ERBB2-dependent signaling induced downstream MAPK cascades and transcriptional programs involving MYC and ELK1 (Fig. 6d), promoting extracellular matrix remodeling and basement membrane degradation, thereby enhancing tumor cell migration and invasive potential [71–74]. We also performed prognostic survival analysis on some cell-type-specific regulators in myeloid cells and stromal cells. High expression levels of STAT2 and YWHAG in myeloid cells, as well as AEBP1 in stromal cells, were significantly associated with poor overall survival in CRC patients (Fig. 6e–g), supporting the clinical relevance of disease-associated regulatory network perturbations identified by CTN. Additional analyses of regulatory perturbations in epithelial cells are presented in Supplementary Figure S8 and Supplementary Note S6.

In addition, for the analysis of lung adenocarcinoma, we first characterized cell type-specific regulators and their functional enrichment in healthy lung tissue (Supplementary Figure S9). We then focused on the differential perturbation network of CAFs in fibroblasts during the development of lung adenocarcinoma, with the dataset composition summarized in Supplementary Table S11, and found that the genes targeted by cell-type-specific regulators were significantly involved in key processes such as tumor microenvironment remodeling and angiogenesis (Supplementary Figure S10 and Supplementary Note S7). In summary, CTN can effectively reveal potential functional mechanisms across multiple cell types in the process of tumor occurrence and development, especially in exploring immune escape, EMT induction and cell-to-cell signal reconstruction, thereby providing new perspectives and theoretical basis for mechanistic studies.

## Discussion

In this study, we proposed CTN, a regulatory network inference method based on bilateral self-representation matrix decomposition, which aims to integrate bulk RNA-seq and scRNA-seq data to reconstruct cell-type-specific GRNs. This method uses bulk RNA-seq data to infer the global gene regulatory context, and extracts cell–cell communication relationships from scRNA-seq data by pseudo-inverse matrix decomposition. On this basis, the specific GRN of each cell type is reconstructed through reverse inference, thereby achieving unified modeling of regulatory directionality and type specificity. CTN demonstrated superior inference performance compared with existing methods in benchmark evaluations, especially in functional pathway reconstruction and key regulator identification. At the same time, CTN can effectively extract functional-specific subnetworks related to key biological processes, identify regulators with high regulatory weights that are not easily detected in traditional differential expression analysis, and reveal their potential role in the process of cell fate determination. In the analysis of tumor development mechanisms, the regulatory network perturbations of myeloid and stromal cells in the CRC microenvironment were systematically analyzed, and key regulators such as STAT2, S100A9, and AEBP1 and their related pathways were clearly identified, and their potential clinical value for prognostic prediction was verified by survival analysis. The CTN method not only has reliable GRN inference capabilities, but also shows broad application prospects in differentiation regulation, disease mechanism analysis and potential target identification.

CTN effectively combines the global regulatory trends provided by bulk RNA-seq with the cell–cell communication patterns reflected in scRNA-seq data by introducing a bilateral self-representation model, significantly improving the robustness of sparse data and the stability of inference results. This method not only enhances the ability to characterize regulatory directionality, but also improves the ability to identify potential heterogeneous communication relationships among cells, providing a more solid foundation for the identification and interpretation of functional modules. Although CTN performs well in many aspects, there are still some areas that deserve further optimization. It should be pointed out that the methods used for inferring a global GRN include, but are not limited to, the three methods mentioned above. Other methods, such as CVP [75], CDD [76], and GNIPLR [77], can be used as alternatives. Any tool that can infer GRN can replace the above three algorithms. Although different GRN tools may have certain differences in network structure and edge strength, they have little impact on the overall performance of CTN in inferring cell-type-specific GRN. In the future, we will consider integrating transcriptome and epigenomic data to construct multimodal regulatory maps to improve the ability to model complex regulatory mechanisms.

The CTN method provides an effective and biologically interpretable solution for the reconstruction of cell-type-specific GRN, achieves high-precision inference of regulatory structures, and provides a powerful tool for a deeper understanding of key factors and regulatory pathways in the process of cell differentiation.

## Methods

### Inference of intercellular communication

In single-cell transcriptome research, the gene expression matrix *R* of size *m× n* is given to represent the expression levels of *m* genes in *n* cells. Previous studies have attempted to extract potential regulatory information from the matrix *R* through the unilateral self-representation model to study gene regulatory relationships [78, 79]. Specifically, the matrix *U* of size *m× m* is obtained by decomposition, as shown in Equation (1):


(1) \begin{eqnarray*} R= UR \end{eqnarray*}

where the matrix *U* represents the GRN adjacency matrix among *m* genes, indicating that the single-cell gene expression data itself contains gene regulatory information.

Similarly, the matrix *R* can also be used to construct a unilateral self-representation model of intercellular communication, as shown in Equation (2):


(2) \begin{eqnarray*} R= RV \end{eqnarray*}

where the matrix *V* describes the adjacency matrix of cell–cell communication among *n* cells, indicating that the single-cell gene expression data itself also contains information about intercellular communication.

However, relying solely on the above two unilateral models cannot fully explore the complex regulatory patterns related to temporal changes in single-cell expression data, especially failing to consider the evolution of cell states in the temporal dimension. Therefore, by combining Equations (1) and (2), the bilateral self-representation model for single-cell gene expression data is constructed [4] to capture the dynamic homeostatic mechanism in the expression process, i.e. cells can maintain the overall steady-state structure even when there are temporal changes when regulating gene expression, which helps to further analyze the gene expression regulation mechanism, as shown in Equation (3):


(3) \begin{eqnarray*} R= URV \end{eqnarray*}

Based on the above theory, we propose a new gene regulation method based on matrix decomposition, CTN, which combines scRNA-seq data and bulk RNA-seq data to construct a cell-type-specific GRN without relying on any prior knowledge, such as TF-target pairs. A GRN can be described as a directed weighted graph connecting different genes (Fig. 1a). The edge from point *i* to point j in the graph represents the regulation of gene ${g}_i$ on gene ${g}_j$, and the weight of the edge reflects the intensity of regulation. The purpose of CTN is to infer gene regulatory relationships based on cell–cell communication relationships, under the assumption that gene regulation information can be obtained from the single-cell gene expression profile itself. CTN constructs a bilateral self-representation model based on the original single-cell gene expression matrix *N* to fully extract gene regulatory relationships and cell–cell communication relationships (Fig. 1a), as shown in Equation (4):


(4) \begin{eqnarray*} N= GNC \end{eqnarray*}

where the left submatrix *G* is the global gene regulatory adjacency matrix inferred from bulk RNA-seq data using the classical gene regulation method, representing the shared regulatory relationships across cell types. The right submatrix *C* represents the cell–cell communication relationships among *n* cells.

Based on the global gene regulation adjacency matrix *G* and the single-cell expression matrix *N*, CTN can obtain the cell–cell communication matrix by solving Equation (5):


(5) \begin{eqnarray*} C={(GN)}^{-1}N \end{eqnarray*}

However, Equation (5) is only applicable when the number of genes and the number of cells are equal and the matrix *GN* is reversible. Because the number of genes and cells often differs in practical applications and the matrix *GN* is generally irreversible, the pseudo-inverse matrix ${(GN)}^{+}$ is used to approximate the solution for matrix *C*, as shown in Equation (6):


(6) \begin{eqnarray*} C={(GN)}^{+}N \end{eqnarray*}

### Inference of global gene regulatory networks

In this study, we constructed the global GRN based on bulk RNA-seq data to provide a shared gene regulatory background for subsequent matrix decomposition-based inference of the cell–cell communication matrix and cell-type-specific GRNs. We use three complementary and widely used GRN inference methods, GENIE3 [7], GENIMS [8], and NARROMI [80], for this purpose. These methods were selected because they are complementary in inference principles: GENIE3 uses tree-based ensemble regression to infer regulatory links, GENIMS adopts a multi-level progressive strategy for large-scale GRN inference, and NARROMI incorporates noise and redundancy reduction strategies to improve network reliability.

The bulk RNA-seq expression matrix *I* is used as input (Fig. 1a), and three initial GRNs are independently constructed using the above three methods. In the integration stage, we adopted a majority-vote strategy inspired by ensemble learning. Specifically, a regulatory edge is retained in the final global GRN only if it is supported by at least two of the three methods, whereas edges supported by only one method are regarded as low-confidence edges and removed. Details of the edge-weight normalization and integration procedures are provided in Supplementary Note S8. This strategy helps integrate complementary evidence from different inference principles and reduce method-specific false-positive and noise-driven edges, thereby improving the stability and interpretability of the global GRN. The influence of different global GRN tool combinations on CTN performance is further evaluated in Supplementary Note S8 and Supplementary Table S7.

### Inference of cell-type-specific gene regulatory networks

After obtaining the cell–cell communication matrix, further analysis is performed based on the cell clustering results or known cell type labels, such as cell type A. Assuming that cell type A contains cells ${c}_{A1},{c}_{A2},\cdots, {c}_{Ak}$, the corresponding gene expression matrix *K* and cell–cell communication submatrix *T* are extracted. A bilateral self-representation model is then constructed for the expression matrix of cell type A (Fig. 1b), as shown in Equation (7):


(7) \begin{eqnarray*} K= SKT \end{eqnarray*}

where the matrix *S* represents the regulatory relationship between specific genes of cell type A, which can be obtained by solving the pseudo-inverse matrix ${(KT)}^{+}$, thereby obtaining the final cell-type-specific GRN of cell type A, as shown in Equation (8):


(8) \begin{eqnarray*} S=K{(KT)}^{+} \end{eqnarray*}

It is worth noting that the element ${S}_{ij}$ represents the value of the *i*-th row and *j*-th column in the matrix *S*. When *i=j*, ${S}_{ij}$ represents the self-regulatory weight of gene ${g}_i$, and when *i≠ j*, ${S}_{ij}$ represents the regulatory weight of gene ${g}_i$ on gene ${g}_j$. For downstream visualization and cross-cell-type comparison, these inferred regulatory weights are converted to absolute values, normalized and scaled to the range of 0–1, and then subjected to sparsity thresholding to remove low-confidence regulatory edges. Details of the sparsity threshold settings are provided in Supplementary Figure S1 and Supplementary Note S1.

Because both the inference of the cell–cell communication matrix and the reconstruction of cell-type-specific GRNs involve pseudo-inverse-based matrix solving, sparse and noisy scRNA-seq data may affect numerical stability. CTN uses the Moore–Penrose pseudo-inverse because the related matrices are often non-square or rank-deficient. In addition, sparsity thresholding and post-inference normalization are applied after matrix solving to reduce the influence of low-confidence edges and noise-driven signals, thereby improving the stability and interpretability of the inferred networks. Additional explicit regularization terms were not introduced in the current implementation because they would require extra hyperparameters and may lead to oversmoothing of biologically meaningful cell-type-specific regulatory differences.

Overall, CTN emphasizes direct modeling of the single-cell expression matrix while incorporating global regulatory constraints derived from bulk RNA-seq data. This design preserves the data-driven nature of the framework while improving the stability and interpretability of the inferred regulatory network, thereby providing a theoretical basis and practical tools for studying cell-type-specific regulatory mechanisms. The computational cost and scalability of CTN are further discussed in Supplementary Note S14.

**Key Points**

- CTN (cell type-specific gene regulatory network) integrates gene regulation and intercellular communication into a bilateral matrix decomposition framework, enabling joint modeling of global regulation and cell communication under tissue homeostasis.
- CTN does not rely on any known regulatory pairs. It uses bulk-derived global gene regulatory networks as constraints and inversely reconstructs cell-type-specific networks via pseudo-inverse matrix decomposition, improving stability and interpretability on sparse, noisy single-cell RNA sequencing data.
- CTN accurately recovers pathway-specific regulatory edges and identifies key regulators with subtle expression changes, revealing regulatory heterogeneity beyond conventional differential analysis.

## Supplementary Material

CTN_Supplementary_bbag525

## Data Availability

The benchmark datasets used in this study are from the NCBI GEO (https://www.ncbi.nlm.nih.gov/geo/) database. The scRNA-seq data are available from GEO accession GSE75748 (hESC), GSE81252 (hHEP), and GSE98664 (mESC). The bulk RNA-seq data are available from GEO accession GSE75748 (hESC), GSE222273 (hHEP), and GSE154572 (mESC). Human hematopoietic differentiation data are obtained from GEO with accession number GSE139369 (scRNA-seq) and GSE74246 (bulk RNA-seq). Human fetal hematopoietic differentiation scRNA-seq data were downloaded from Zenodo https://zenodo.org/record/7879228 [81], bulk RNA-seq data are obtained from GEO with accession number GSE122982. CRC data are available from GEO accession GSE144735 (scRNA-seq), bulk RNA-seq data are obtained from the TCGA (http://cancergenome.nih.gov) database. Details of the input datasets are provided in Supplementary Note S11. The source code of the CTN algorithm is available at https://github.com/xykxingchen/CTN.
