# Supplementary material for: Constructing cell-type-specific gene regulatory networks from cell–cell communication and a global gene regulatory network
Source: Brief Bioinform. 2026 Sep 26;27(5):bbag525. doi: 10.1093/bib/bbag525 (PMC13615534; doi:10.1093/bib/bbag525)
Supplement: Supplementary file 1 [file ctn_supplementary_bbag525.docx]

**Constructing cell type-specific gene regulatory networks from cell-cell communication and a global gene regulatory network**

**Supplementary Notes**

**Supplementary Note 1. Details of CTN sparsity constraint settings**

Considering the inherent sparsity of cell-cell communication and gene regulatory relationships in biological systems, we introduced two key sparsity constraint values for the intercellular communication adjacency matrix obtained using the bilateral self-representation model and the final cell type-specific GRN adjacency matrix. Constraint value $x$ is used to control the sparsity of intercellular communication, and the constraint value $y$ is used to regulate the sparsity of cell type-specific GRNs. Specifically, in the corresponding directed weighted graph, an edge is considered valid only when its weight exceeds the corresponding threshold. We systematically evaluated how different combinations of these two constraint values affected the final prediction performance.

We traversed the value combinations of constraint values $x$ and $y$, and calculated the AUROC value that measures the prediction accuracy. The results showed that in the hESC data, the constraint value range with the best overall performance was stable at $x\in[0.2, 0.5]$, $y\in[0.5, 0.7]$ (Supplementary Figure 1A). In the hHEP data, the constraint value range with the best overall performance was stable at $x=0.5$, $y\in[0.5, 0.8]$ (Supplementary Figure 1B). In the mESC data, the constraint value range with the best overall performance was stable at $x\in[0.1, 0.2]$, $y\in[0.4, 0.6]$ (Supplementary Figure 1C). Taking into account the differences in cell-cell communication and gene regulation mechanisms between species, this study ultimately determined that the optimal range of the two constraint values settings in the human dataset is $x\in[0.4, 0.5]$, $y\in[0.6, 0.7]$, and in the mouse dataset it is $x\in[0.1, 0.2]$, $y\in[0.4, 0.6]$.

Because gold-standard cell type-specific GRNs are not available for real disease datasets such as CRC and LUAD, the sparsity parameters cannot be directly re-optimized using AUROC or AUPRC in these datasets. To avoid overfitting or circular interpretation based on downstream biological observations, we used the stable high-performance parameter ranges identified from benchmark datasets and applied the corresponding species-matched settings to real disease datasets.

To further assess the applicability of these parameters in real disease datasets, we evaluated the biological consistency of the inferred networks. In CRC and LUAD datasets, known marker genes of major cell types showed significantly higher out-degree in their corresponding cell type-specific GRNs than randomly selected control genes (p < 0.01), and enrichment analysis of cell type-specific regulators revealed biological processes consistent with the expected functions of the corresponding cell types. These observations indicate that the inferred networks preserve meaningful cell type-specific regulatory features under the selected sparsity settings, providing additional support for the use of benchmark-derived parameters in real disease analyses.

**Supplementary Note 2. Details of GRN inference methods and parameter settings**

To verify the effectiveness of the cell type-specific GRN constructed by CTN, this study used the unified benchmark datasets and systematically compared with five classic GRN inference methods. For methods requiring parameter settings, we followed the default values recommended in the original publications.

Based on feature selection and ensemble tree models, GENIE3 decomposes the GRN inference problem into multiple independent regression problems to predict gene expression patterns, and finally outputs a ranked list of regulatory relationships between genes.

GENIMS adopts a three-layer progressive strategy. Feature selection is performed based on guided regularized random forests to solve individual regression problems, then norm normalization is used to reduce bias, and finally, given the inherent sparsity of GRN, the preliminary results are refined. This method involves two key parameters, namely the global penalty parameter $\gamma$ and the normalization parameter $q$. In this study, $\gamma=0.7$ and $q=3$ are set for comparison.

PPCOR is an R package that quantifies the strength of regulatory relationships between gene pairs by calculating partial correlation coefficients or semi-partial correlation coefficients. Among them, the partial correlation coefficient measures the net correlation between two genes after excluding the influence of other genes. The semi-partial correlation coefficient measures the correlation between the independent contribution of the regulatory factor and the expression of the target gene after excluding the influence of other regulatory factors on the target gene. Semi-partial correlation analysis was used in this study to quantify the independent contribution of each regulatory factor to the expression of the target gene, thereby effectively avoiding the interference of co-regulatory factors.

DeepSEM is a deep generative model that infers regulatory relationships by jointly modeling GRN structure and transcriptome data. Its core mechanism is to design a two-layer neural network architecture containing GRN layer and inverse GRN layer to reveal the underlying gene regulatory logic.

SLIVER integrates causal structural equation model and graph decomposition, and introduces a set of low-dimensional factor nodes to integrate the regulatory relationship between genes according to gene function. The method requires the input of original scRNA-seq data and its corresponding benchmark network.

For the global GRN construction used in CTN, the parameter settings of the three GRN inference methods were as follows. GENIE3 was run using the default parameters of the GENIE3 implementation, with the number of threads set to 10 for parallel computation. For GENIMS, the parameters were set as $\gamma=0.7$ and $q=3$. For NARROMI, the regularization parameter $\lambda$ was set to 1, and the MI filtering threshold was set to 0.05. In our implementation, the RO-related threshold parameter was set to 0.05, and the integration weight controlling the contribution of RO in the final MI–RO integration was set to 0.6.

**Supplementary Note 3. Analysis of LMP branch in human fetal hematopoietic differentiation**

It is known that the downstream differentiated cell types of the LMP branch include B cells, monocytes, and plasmacytoid dendritic cells (pDCs). By comparing monocytes with B cells (Supplementary Figure 4A) and monocytes with pDCs (Supplementary Figure 4B), we identified several candidate lineage-specific regulators that play specific regulatory roles in monocytes: SP1, NFKB1, and ANXA11 (Supplementary Table 5). These lineage-specific regulators showed higher logFC values in the differential regulation analysis and had a higher overall regulatory weight in monocytes (Supplementary Figure 4C). Furthermore, we extracted subnetworks centered on these lineage-specific regulators from the cell type-specific GRN of monocytes (Supplementary Figure 4D) and found that the regulated target genes play roles in the differentiation process of monocytes. For example, MECP2 and NCBP2 (Supplementary Figure 4D) are essential for maintaining monocyte homeostasis and differentiation process [1, 2]. IRF3 (Supplementary Figure 4D) plays a key role in monocyte hematopoietic development and maturation [3].

Similarly, by comparing pDCs with B cells (Supplementary Figure 5A) and pDCs with monocytes (Supplementary Figure 5B), we also identified candidate lineage-specific regulators BDP1, AFF4, STAT1, and TCF4, which specifically play regulatory roles in pDCs (Supplementary Table 6). These lineage-specific regulators have a higher regulatory weight in pDCs (Supplementary Figure 5C). Studies have found that both functional isoforms of TCF4 are expressed in pDCs, of which the "long" TCF4 isoform shows pDC specificity and regulates pDC development in vivo [4]. By extracting the regulatory subnetwork centered on lineage-specific regulators of pDCs (Supplementary Figure 5D), we found that the target gene BCL11A has been identified as an important lineage-specific regulator of pDC development (Supplementary Figure 5D). BCL11A loss will lead to interference with progenitor cell generation and loss of downstream pDC cell lineages [5].

**Supplementary Note 4. Analysis of cell type-specific regulators in normal colorectal tissue**

In B cells (Figure 5A), CD79A/CD79B, as an important component of B-cell receptor (BCR), plays an indispensable role in maintaining BCR function, signal initiation and transduction, and is involved in regulating a variety of important physiological processes [6]. In addition, MZB1 has been confirmed to be a marker gene for healthy B cells [7]. In epithelial cells (Figure 5A), EPCAM, SLC26A2 and TFF1 are widely recognized as marker genes. S100P protein is highly active in the colonic epithelium [7-9]. CLDN7 maintains the homeostasis of intestinal stem cells by regulating multiple signaling pathways, thereby supporting the continuous self-renewal and repair of intestinal epithelial cells. At the same time, specific loss of CLDN7 in intestinal epithelial cells may lead to the occurrence of spontaneous colitis [10]. S100A9 and ACTA2 (Figure 5A) have been confirmed to be marker genes for myeloid cells and stromal cells, respectively [7]. In T cells (Figure 5A), LAT plays an important role in T cell activation [11]. The protein encoded by CD3D is part of the T-cell receptor/CD3 complex and is involved in T cell development and signal transduction [12]. In addition, PSMB9 is also recognized as a marker gene for T cells [13]. The above results indicate that different cell populations rely on their own unique regulatory networks to maintain their specific functions under steady state.

**Supplementary Note 5. Functional pathway enrichment analysis of cell type-specific regulators in normal colorectal tissue**

To characterize the functional relevance of cell type-specific regulators identified by CTN, we performed biological process enrichment analysis separately for regulators from each major cell population. The enrichment results for myeloid and stromal cell specific regulators are shown in Figure 5B and Figure 5C, respectively, while the corresponding results for B cells, T cells, and epithelial cells are presented in Supplementary Figure 6A-6C.

The analysis revealed that regulators from different cell types are enriched in distinct biological processes closely related to the maintenance of cellular homeostasis and execution of cell type-specific functions. In myeloid cells, regulators are predominantly enriched in immune-related processes, including regulation of immune effector processes (Figure 5B), consistent with the central role of myeloid cells in innate immune responses. In stromal cells, regulators are mainly enriched in pathways associated with vasculature development and angiogenesis (Figure 5C), reflecting the essential contribution of stromal cells to vascular structure and function within the tissue microenvironment [14]. Consistent patterns were observed in other cell types. In B cells, enrichment of Fc-epsilon receptor-related processes (Supplementary Figure 6A) is consistent with the established role of B cells in immune regulation, growth, and differentiation [15]. In T cells, enrichment of immune effector regulation processes (Supplementary Figure 6B) highlights the role of regulatory T cell populations in maintaining intestinal immune tolerance through dynamic control of immune responses [16]. In epithelial cells, regulators are enriched in biological processes related to oxidative stress and endoplasmic reticulum stress responses (Supplementary Figure 6C), both of which are critical for maintaining intestinal epithelial barrier homeostasis and are closely associated with inflammatory bowel disease and colorectal cancer [17-19].

**Supplementary Note 6. Epithelial cell-specific GRN variation in colorectal cancer and normal tissue**

Epithelial cells play a critical role in colorectal cancer (CRC) progression by driving epithelial-mesenchymal transition (EMT), tumor invasion, and metastasis [20, 21]. Comparison of epithelial cell type-specific GRNs between healthy and CRC samples revealed pronounced disease-associated network perturbations (Supplementary Figure 8A). Regulators with high network centrality and strong disease-specific regulatory changes were prioritized, leading to the identification of HES6, S100P, and TFF1 as key perturbed regulators in epithelial cells.

Functional enrichment analysis of downstream target genes showing significant regulatory differences highlighted EMT-related biological processes (Supplementary Figure 7C), with the mTOR signaling pathway emerging as one of the most significantly enriched pathways (Supplementary Figure 8B). Pathway reconstruction showed that differentially regulated genes converge on the mTOR signaling axis, which integrates growth factor signaling, nutrient status, and environmental cues. Activation of the PIK3CA-AKT pathway promotes mTORC2-dependent AKT activation through scaffold proteins RICTOR and PRR5, while increased MAPKAP1 further enhances mTORC2 activity (Supplementary Figure 8B). Activated AKT suppresses TSC2, thereby activating mTORC1 and sustaining a positive feedback loop that promotes the expression of EMT-related transcriptional programs [22]. In addition, chaperone proteins such as TELO2 and TTI1 support the stability and function of mTOR complexes (Supplementary Figure 8B). Together, these findings indicate that coordinated inhibition of mTORC1 and mTORC2 may represent a potential strategy to suppress EMT and tumor invasion in CRC.

**Supplementary Note 7. Analysis of the potential regulatory mechanism of LUAD**

In order to better illustrate the application value of CTN in revealing the potential regulatory mechanisms in the development of diseases, this study also focused on lung adenocarcinoma (LUAD) to construct a differential perturbation network under LUAD disease conditions. Prior to this, it is also necessary to identify the key regulatory modules and core functional genes of each cell type in the healthy group under steady-state conditions to provide a reliable reference baseline for the comparison of network perturbations under subsequent disease conditions.

This study integrated scRNA-seq data (Supplementary Table 11) of human LUAD [23] and bulk RNA-seq data from The Cancer Genome Atlas (TCGA). Based on the cellular composition of healthy adjacent tissues and tumor core regions, samples were divided into healthy and diseased groups. Cell type-specific GRNs were constructed using expression profiles from the healthy group, and systematic comparisons across cell types were performed to identify regulators with pronounced cell type-specific regulatory activity. We identified a set of regulators exhibiting both strong regulatory weights and distinct cell type-specific expression patterns. Supplementary Figure 9A presents representative regulators from different cell populations, illustrating the cell type specificity and regulatory weight revealed by the CTN framework. These regulators provide a foundation for subsequent analyses of disease-associated regulatory perturbations. In B cells (Supplementary Figure 9A), CD53 has been reported to be closely associated with B cell development and immune function [24], while FCGR2B encodes an inhibitory Fc receptor that maintains immune homeostasis by preventing excessive B cell activation [25]. In natural killer (NK) cells (Supplementary Figure 9A), CD300A is widely expressed and mediates inhibitory signaling following ligand engagement [26], whereas KLRD1 is highly expressed and plays a central role in NK cell-mediated cytotoxic responses and anti-tumor immunity [27]. In T cells (Supplementary Figure 9A), DOK2 expression is significantly upregulated during T cell maturation, indicating an important role in T cell differentiation [28].

To further characterize the biological functions of cell type-specific regulators identified in healthy lung tissue, functional enrichment analysis was performed for regulators from each cell type, including the representative regulators shown in Supplementary Figure 9A. Biological process enrichment was conducted separately for each cell population to assess whether the identified regulators converge on cell type-specific functional programs. The enrichment results are summarized in Supplementary Figure 9B-9E. The analysis revealed that regulators from different cell types were significantly enriched in biological processes related to cellular homeostasis maintenance and specialized functional execution. Specifically, regulators in B cells were enriched in immunoglobulin mediated immune responses (Supplementary Figure 9B), regulators in NK cells were enriched in defense responses to fungal infection (Supplementary Figure 9C), regulators in T cells were enriched in cytokine receptor binding and immune effector processes (Supplementary Figure 9D), and regulators in fibroblasts were enriched in collagen biosynthetic processes (Supplementary Figure 9E). These enrichment patterns are consistent with well-established biological roles of the corresponding cell populations in lung tissue. B cells serve as the exclusive sources of immunoglobulin production [29]. Fibroblasts maintain tissue structural integrity through collagen synthesis and extracellular matrix deposition, processes that are tightly regulated to preserve lung homeostasis [30]. NK cells contribute to pulmonary immune defense by mediating cytotoxic responses and secreting cytokines and chemokines that activate additional immune components [31], while T lymphocytes function as central regulators of cellular immunity by orchestrating cytokine-mediated immune responses [32]. Together, these results demonstrate that CTN identifies biologically meaningful, cell type-specific regulators in LUAD, and that the regulatory networks constructed from healthy tissues capture core regulatory features characteristic of each immune cell population, providing a robust reference for downstream analyses of disease-related network alterations.

To characterize disease-associated regulatory perturbations in lung adenocarcinoma (LUAD), regulatory edge weights were systematically compared between healthy and disease states across cell type-specific GRNs. On this basis, we comprehensively analyzed the dynamic changes in network regulatory weights, aiming to reveal the core molecular mechanisms driving the occurrence and development of LUAD. Given the important role of cancer-associated fibroblasts (CAFs) in the tumor microenvironment, especially in promoting tumor cell invasion and angiogenesis by secreting growth factors such as VEGF, and proinflammatory factors, we focused on changes in fibroblast regulatory network weights between healthy and LUAD states to identify potential targets for anti-tumor immunotherapy. Regulators were ranked according to both network centrality and the magnitude of disease-associated changes in regulatory edge weight. TMEM160 and CLEC11A exhibited the largest increases in regulatory connectivity and the most pronounced perturbations between healthy and LUAD states, and were therefore selected as core regulators in the fibroblast perturbation network (Supplementary Figure 10A). TMEM160 has been implicated in multiple cancer-related processes, including cell proliferation, migration, and resistance to radiotherapy and chemotherapy [33]. CLEC11A has been reported to enhance angiogenic responses induced by VEGF-A and basic fibroblast growth factor [34], suggesting a potential role in tumor-associated angiogenesis. To further investigate the functional consequences of fibroblast-specific regulatory perturbations, pathway enrichment analysis was performed on all genes contained in the TMEM160 and CLEC11A centered perturbation network. The enrichment results identified the HIF-1 signaling pathway as a key pathway associated with LUAD progression (Supplementary Figure 10B). Reconstruction of the HIF-1 signaling pathway revealed coordinated regulation of angiogenesis-related programs (Supplementary Figure 10B). Upstream MAP2K1-MAPK3 and PIK3R1-AKT1 signaling converged on activation of RPS6KB2 (Supplementary Figure 10B), which promoted RPS6-mediated upregulation of HIF1A. Under hypoxic conditions, HIF1A cooperated with ARNT to activate downstream angiogenic factors, including VEGF and ANGPT2 (Supplementary Figure 10B), thereby driving angiogenesis and vascular remodeling. In addition, prognostic survival analysis demonstrated that elevated expression levels of fibroblast-specific regulators TMEM160 and CLEC11A were significantly associated with poorer survival outcomes in LUAD patients (Supplementary Figure 10C-10D), supporting the clinical relevance of fibroblast-centered regulatory perturbations identified by CTN.

LUAD data are available from GEO accession GSE131907 (scRNA-seq), bulk RNA-seq data are obtained from the TCGA database. To investigate differences in GRNs between normal and disease groups, data from both normal and disease groups are processed uniformly. The scRNA-seq data includes four cell types: B cells, fibroblasts, NK cells and T cells. The corresponding bulk RNA-seq data includes 24 samples for both the normal and disease groups. All data are quality-controlled and normalized, retaining 8182 common genes. The number of cell types in each group is listed in Supplementary Table 11. The resulting data sets are: the normal group includes scRNA-seq data from four cell types, 8731 cells, and 8182 genes, as well as bulk RNA-seq data from 24 samples and 8182 genes; the disease group includes scRNA-seq data from four cell types, 12458 cells, and 8182 genes, as well as bulk RNA-seq data from 24 samples and 8182 genes.

**Supplementary Note 8. Details for** **global GRNs**

For global GRN construction in CTN, GENIE3, GENIMS and NARROMI were first used to independently infer adjacency matrices from the bulk RNA-seq expression matrix. Since the edge scores produced by different algorithms are not directly comparable, the edge weights from each method were transformed and normalized before integration. Specifically, the absolute values of the inferred edge scores were used, followed by method-specific scaling, sigmoid transformation and min-max scaling to normalize each adjacency matrix to the range of 0–1.

The normalized matrices were then integrated using a majority-vote strategy. For each candidate regulatory edge, the edge was retained in the final global GRN only when it was supported by at least two of the three methods. Edges supported by only one method were set to 0. For retained edges, the final edge weight was calculated by aggregating the normalized scores from the three methods using a $p$-norm-based scoring scheme. Specifically, let $G^{GENIE3}$, $G^{GENIMS}$, and $G^{NARROMI}$ denote the normalized adjacency matrices inferred by GENIE3, GENIMS, and NARROMI, respectively. For each retained regulatory edge, its integrated weight in the final global GRN was calculated as:

$$G_{ij}^{\mathrm{global}}=\frac{\left[ \left( G_{ij}^{GENIE3} \right)^{p}+\left( G_{ij}^{\mathrm{GENIMS}} \right)^{p}+\left( G_{ij}^{\mathrm{NARROMI}} \right)^{p} \right]^{1/p}}{3},\quad p=2$$

Here, $G_{ij}^{global}$represents the integrated weight of the regulatory edge from gene $i$ to gene $j$ in the final global GRN. This integration strategy reduces method-specific noise and false-positive edges while preserving regulatory relationships consistently supported by multiple inference algorithms.

To evaluate the impact of global GRN construction on CTN performance, we tested different combinations of GRN inference methods. Specifically, we replaced one of the three methods used in the main analysis with PPCOR [35], and systematically evaluated the accuracy of cell type-specific GRNs inferred by CTN using different global GRN inputs on benchmark datasets from two species, hESC and mESC. Although performance differences were observed among different combinations, the overall performance variation was limited (Supplementary Table 7), indicating that CTN performance was not highly dependent on a single specific global GRN inference tool.

Further comparison showed that combination of multiple GRN inference methods generally outperformed single-method settings. Compared with using a single GRN inference method, integrating multiple methods improved AUROC by approximately 4% in human data and nearly 10% in mouse data. Among the tested combinations, the combination of GENIE3, GENIMS, and NARROMI achieved the best performance across multiple evaluation metrics.

In summary, we recommend that the combined results of multiple GRN inference tools should be used preferentially in CTN analysis to reduce method-specific errors and improve the robustness of network inference. Users may also provide their own preconstructed global GRN as input to the CTN framework according to specific research needs.

**Supplementary Note 9. Benchmark datasets and evaluation metrics**

This study uses three scRNA-seq datasets and their corresponding tissue-derived bulk RNA-seq data to systematically evaluate the performance of the CTN method in inferring cell type-specific GRNs. The specific sources and detailed descriptions of each dataset are described below.

The hESC dataset [36] contains scRNA-seq data of 758 cells at 0, 12, 24, 36, 72, and 96 h, as well as bulk RNA-seq data at the corresponding time points, analyzing the dynamic transcriptome changes during the differentiation of hESCs into definitive endoderm cells. The hHEP dataset [37] records scRNA-seq data from 425 cells at multiple time points during the differentiation of induced pluripotent stem cells (iPSCs) into hepatocyte-like cells. The corresponding bulk RNA-seq data [38] come from the resulting hepatoid organoids, which are composed of multiple liver-related cell types and derived from human iPSCs. The mESC dataset [39] contains scRNA-seq time course data of 421 mESCs during their differentiation into primitive endoderm cells. The corresponding bulk RNA-seq data [40] are time course data of mESCs during directed differentiation. All the above datasets can be obtained at the ‘Data and code availability’ section.

To comprehensively evaluate the performance of CTN and other algorithms in the task of reconstructing GRNs, we used AUROC and AUPRC as the main evaluation indicators, and compared the inferred network with the real network from the gold standard dataset.

AUROC is used to measure the overall ranking performance of the model's ability to distinguish between positive and negative samples. It focuses on the model's discrimination ability in the full sample range and can provide a global performance benchmark. AUPRC, on the other hand, focuses more on the model's recognition accuracy of positive samples (true regulatory relationships) at different recall levels and is suitable for scenarios with extremely unbalanced categories. In the GRN inference task, since the true regulatory relationship is extremely rare among all possible gene pairs and the ratio of positive and negative samples is seriously unbalanced, AUPRC becomes a more discriminative and representative key indicator, which can directly reflect the accuracy and practical value of the model's Top-k edge prediction. However, AUROC is still a necessary supplement to reflect the overall ranking performance of the model and provide a reference baseline for performance evaluation.

Combining the two indicators can complementarily evaluate the model performance from the two dimensions of local accuracy and global ranking, effectively alleviating the evaluation bias caused by data imbalance.

**Supplementary Note 10. Extract the real network from the benchmark dataset**

The true network is extracted by first selecting all genes from the benchmark dataset that are included in the cell type-specific GRNs. KEGG enrichment analysis is then performed to identify biological pathways that play key roles in the differentiation of the corresponding cell types. The KEGG analysis helps pinpoint the critical molecular mechanisms involved in cell differentiation and the genes associated with these pathways. Function-specific subnetworks are subsequently constructed by extracting genes that participate in differentiation-related pathways. These subnetworks represent the regulatory relationships between genes involved in cell differentiation processes. Finally, the extracted regulatory relationships are compared to the ground-truth GRN provided by the benchmark dataset to ensure accurate representation of gene regulatory interactions. The function-specific subnetworks serve as the true network and are used for comparing predicted networks. The accuracy of predicted regulatory edges within these subnetworks is assessed by calculating AUROC, comparing the predicted edges to the corresponding true network from the benchmark dataset.

**Supplementary Note 11. Input datasets**

This study is based on three high-throughput transcriptome datasets and uses the CTN method to construct different GRNs to reveal potential regulatory mechanisms. Each dataset contains scRNA-seq and its corresponding bulk RNA-seq data, all of which have been preprocessed, quality controlled, and normalized.

**(1) Human hematopoietic differentiation data**

The original dataset contains 13 hematopoietic-related cell types, totaling 11097 genes. This study focuses on five of these cell types and converts the original expression data into TPM format. After quality control and normalization, 10905 genes were retained, and the number of cells in each cell type is shown in the (Supplementary Table 8). The corresponding bulk RNA-seq data contains 29 samples, and the expression data is also converted to TPM format and normalized, retaining 21696 genes. After classifying and quality controlling the data of each cell type, 4455 common genes with the bulk RNA-seq data were retained. Finally, scRNA-seq data containing 5 cell types, a total of 10226 cells and 4455 genes, and bulk RNA-seq data containing 29 samples and 4455 genes were obtained as input.

**(2) Human fetal hematopoietic differentiation data**

This dataset contains 11 cell types in 3 hematopoietic differentiation branches. This study focuses on the analysis of the LMP branch (monocyte, pDC, B cell) and the MEMP branch (erythroid cell, mast cell, megakaryocyte). The original scRNA-seq data of each cell type were classified and sorted to retain 5239 common genes. The number of each cell type is shown in the (Supplementary Table 9). The corresponding original bulk RNA-seq data contains 12 samples and 58051 genes. After gene name conversion and deduplication, 34157 genes are retained. After quality control of the data of each cell type, 5034 common genes between the bulk RNA-seq data are retained. Finally, scRNA-seq data containing 11 cell types, a total of 4017 cells and 5034 genes, and bulk RNA-seq data containing 12 samples and 5034 genes were obtained as input.

**(3) Colorectal cancer data**

To study the differences in GRN between the normal group and the diseased group, this study uniformly processes the data of the normal group and the diseased group. The scRNA-seq data contains 5 cell types: B cell, epithelial cell, myeloid cell, stromal cell, and T cell. In the corresponding bulk RNA-seq data, the number of samples in the normal group and the diseased group is 24. All data were quality controlled and normalized to retain 8504 common genes. The number of cell types in each group is listed in (Supplementary Table 10). Finally, two groups of data were obtained, the normal group contained 5 cell types, a total of 9443 cells, 8504 genes of scRNA-seq data, and 24 samples, 8504 genes of bulk RNA-seq data, and the diseased group contained 5 cell types, a total of 7930 cells, 8504 genes of scRNA-seq data, and 24 samples, 8504 genes of bulk RNA-seq data.

**Supplementary Note 12. Expression, regulation specificity**

In order to evaluate the cell type specificity of each regulator $i$ and its regulatory weight in different cell types $j$, this study first calculated the average regulatory weight $w_{ij}$ of the regulator in each cell type. Subsequently, in order to eliminate the overall difference in regulatory weight between different cell types, the weights within each cell type are normalized, and the relative weight $\bar{w}_{ij}=w_{ij}/\sum_{i} w_{ij}$ is calculated. Based on the relative weight, the cell type-specific score $s_{ij}=\bar{w}_{ij}/\sum_{j} \bar{w}_{ij}$ of regulator $i$ in different cell types $j$ is further calculated.

Using the same computational framework, we further evaluate the cell type-specific expression level based on gene expression data. Specifically, the average regulatory weight $w_{ij}$ used in the above calculation is replaced with the average expression of the gene in each cell type to obtain the cell type-specific score at the expression level. In this study, cell type-specific regulators are defined as regulators with the regulatory specificity score $s_{ij}$ greater than 0.5 in the specific cell type and no significant cell specificity at the gene expression level.

**Supplementary Note 13. Differential regulation and differential expression logFC values**

To facilitate comparative analysis of regulatory activity and gene expression between different cell types, we define the difference index logFC as the difference between the two cell types in ${log}_{2}(x+1)$. The variable $x$ represents the regulatory weight in regulatory activity analysis or the gene expression in gene expression analysis. In this study, the absolute value of logFC value greater than 1.2 is set as the threshold to identify regulators or differentially expressed genes with significant differences.

**Supplementary Note 14. Computational cost of CTN**

The computational cost of CTN mainly arises from two pseudo-inverse-based matrix-solving steps. The first step is the inference of the cell-cell communication matrix $C$. For a single-cell expression matrix $N$ with $m$ genes and $n$ cells, the dominant computational cost of this step can be approximated as $O(m\times n^{2})$. The second step is the reconstruction of cell type-specific GRNs. For the $i$-th cell type containing $k_{i}$ cells, the corresponding computational cost can be approximated as $O(m\times k_{i}^{2})$, and the total cost across all cell types is $\sum O(m\times k_{i}^{2})$. Therefore, the overall running time of CTN mainly depends on the number of genes, the total number of cells, and the distribution of cells across different cell types. For a typical dataset with approximately 5,000 genes and 10,000 cells, a complete CTN run usually requires approximately 15–25 minutes, depending on matrix density, preprocessing steps, hardware configuration, and numerical implementation.

The memory requirement of CTN is mainly determined by the storage of the single-cell expression matrix $N$, the global GRN matrix $G$, the cell-cell communication matrix $C$, cell type-specific GRN matrices $S$, and several intermediate matrices generated during pseudo-inverse calculation and network reconstruction. For a typical dataset with approximately 5,000 genes and 10,000 cells, CTN typically requires 8–12 GB of memory. For very large datasets, such as those with more than 50,000 cells, the dense cell-cell communication matrix may become the major memory bottleneck. Therefore, gene filtering, sparse storage after thresholding, cell-type-wise analysis, or downsampling strategies may be considered to improve scalability.

**Supplementary Figures**

**Supplementary Figure 1. CTN method constraint values setting range.** AUROC values of constraint values $x$ and $y$ in (A) hESC, (B) hHEP, and (C) mESC data.

**Alt text:** AUROC-based parameter analysis showing the performance of different CTN sparsity constraint settings in hESC, hHEP, and mESC benchmark datasets.

**Supplementary Figure 2. Analysis of cell type-specific GRNs in human hematopoietic differentiation datasets.** (A) Specific values of cell type-specific regulators at the gene expression level. (B) Cell type-specific GRNs in erythroid cells. (C) Pathways involved in cell type-specific regulators and regulated target genes in erythroid cells.

**Alt text:** Supplementary analysis of human hematopoietic differentiation, showing expression-level specificity of cell type-specific regulators, the erythroid regulatory network, and pathways involving erythroid regulators and target genes.

**Supplementary Figure 3. Enrichment analysis of B cells and erythroid cells in human fetal hematopoietic differentiation.** (A) Human fetal hematopoietic cell differentiation lineage diagram. (B) Bubble diagram of B cell-related pathway enrichment analysis. (C) Bubble diagram of erythroid cell-related pathway enrichment analysis.

**Alt text:** Human fetal hematopoietic lineage diagram and enrichment analyses showing biological pathways associated with B cell and erythroid cell regulatory subnetworks.

**Supplementary Figure 4. Analysis of lineage-specific regulators in monocytes during human fetal hematopoietic differentiation.** By comparing monocytes with B cells (A) and monocytes with pDCs (B) cell type-specific GRNs, we obtained lineage-specific regulators that affect monocyte differentiation. (C) The regulatory weights of these regulators are relatively high in monocytes. (D) Regulatory network composed of lineage-specific regulators and target genes.

**Alt text:** Analysis of monocyte lineage-specific regulators in fetal hematopoiesis, showing comparisons with B cells and plasmacytoid dendritic cells, higher regulatory weights in monocytes, and a monocyte regulator-target network.

**Supplementary Figure 5. Analysis of lineage-specific regulators of pDCs in human fetal hematopoietic differentiation.** By comparing pDCs with B cells (A) and pDCs with monocytes (B) cell type-specific GRNs, we obtained the lineage-specific regulators that affect pDCs differentiation. (C) The regulatory weights of these regulators are relatively high in pDCs. (D) The regulatory network composed of lineage-specific regulators and target genes.

**Alt text:** Analysis of plasmacytoid dendritic cell lineage-specific regulators in fetal hematopoiesis, showing comparisons with B cells and monocytes, higher regulatory weights in plasmacytoid dendritic cells, and a plasmacytoid dendritic cell regulator-target network.

**Supplementary Figure 6. Biological processes involved in genes in cell type-specific regulatory networks in normal colorectal tissue.** (A) B cells. (B) T cells. (C) Epithelial cells.

**Alt text:** Enrichment analysis of genes within cell type-specific regulatory networks from normal colorectal tissue, including B cells, T cells, and epithelial cells.

**Supplementary Figure 7. Enrichment analysis of target genes showing significant regulatory differences in disease states.** (A) Myeloid cells. (B) Stromal cells. (C) Epithelial cells.

**Alt text:** Enrichment analysis of target genes with significant disease-associated regulatory differences in myeloid cells, stromal cells, and epithelial cells.

**Supplementary Figure 8. Differential perturbation regulatory network analysis of epithelial cells in CRC.** (A) Differential changes in GRN between normal and disease groups. (B) Main branches of the mTOR signaling pathway involved in perturbation network genes in epithelial cells.

**Alt text:** Analysis of epithelial cell regulatory perturbations in colorectal cancer, showing differential gene regulatory network changes and mTOR signaling pathway branches involving perturbed network genes.

**Supplementary Figure 9. Biological processes involved in genes in cell type-specific regulatory networks in normal lung tissue.** (A) Specificity index of specific regulators for each cell type. Biological processes involved in genes in cell type-specific regulatory networks of (B) B cells, (C) NK cells, (D) T cells, (E) Fibroblasts.

**Alt text:** Analysis of normal lung tissue, showing cell type-specific regulators and enriched biological processes in B cells, natural killer cells, T cells, and fibroblasts.

**Supplementary Figure 10. Differential perturbation regulatory network analysis of fibroblasts in LUAD.** (A) Differential changes in GRN between normal and disease groups. (B) Main branches of HIF-1 signaling pathway involved in perturbation network genes in fibroblasts. Prognostic survival analysis of regulators CLEC11A (C) and TMEM160 (D).

**Alt text:** Analysis of fibroblast regulatory perturbations in lung adenocarcinoma, showing differential gene regulatory network changes, HIF-1 signaling pathway involvement, and survival analysis involving CLEC11A and TMEM160.

**Supplementary Tables**

**Supplementary Table 1. The specific values of the metrics of each method**

|  | hESC | | hHEP | | mESC | |
| --- | --- | --- | --- | --- | --- | --- |
|  | AUROC | AUPRC | AUROC | AUPRC | AUROC | AUPRC |
| CTN | **0.611** | **0.323** | **0.575** | **0.398** | **0.664** | **0.402** |
| GENIE3 | 0.546 | 0.270 | 0.507 | 0.336 | 0.613 | 0.375 |
| GENIMS | 0.502 | 0.249 | 0.508 | 0.344 | 0.590 | 0.385 |
| PPCOR | 0.550 | 0.223 | 0.503 | 0.331 | 0.555 | 0.271 |
| DeepSEM | 0.534 | 0.268 | 0.517 | 0.344 | 0.580 | 0.370 |
| SLIVER | 0.524 | 0.257 | 0.529 | 0.365 | 0.572 | 0.262 |

**Supplementary Table 2. Regulatory specificity of cell type-specific regulators in human hematopoietic differentiation data**

| Genes | HSC | Erythroid | GMP | Monocyte | CLP |
| --- | --- | --- | --- | --- | --- |
| ETV6 | 1.0000 | 0.0000 | 0.0000 | 0.0000 | 0.0000 |
| FLI1 | 0.8609 | 0.0373 | 0.1018 | 0.0000 | 0.0000 |
| MLLT10 | 0.9943 | 0.0003 | 0.0000 | 0.0000 | 0.0054 |
| PLAGL1 | 0.8948 | 0.0000 | 0.1052 | 0.0000 | 0.0000 |
| NR3C1 | 0.7812 | 0.0035 | 0.0522 | 0.1448 | 0.0183 |
| LMO2 | 0.6327 | 0.2085 | 0.0823 | 0.0474 | 0.0292 |
| DDIT3 | 0.5879 | 0.1734 | 0.1191 | 0.0778 | 0.0418 |
| FIP1L1 | 0.5530 | 0.0000 | 0.1902 | 0.0131 | 0.2437 |
| HP1BP3 | 0.5267 | 0.0930 | 0.0020 | 0.1857 | 0.1926 |
| E2F4 | 0.2894 | 0.5486 | 0.1043 | 0.0171 | 0.0405 |
| DNMT1 | 0.3180 | 0.5265 | 0.1486 | 0.0062 | 0.0007 |
| ATF6 | 0.0123 | 0.9877 | 0.0000 | 0.0000 | 0.0000 |
| NR1H2 | 0.0059 | 0.9401 | 0.0000 | 0.0538 | 0.0001 |
| ADNP | 0.1306 | 0.7004 | 0.0418 | 0.0006 | 0.1267 |
| DBP | 0.0924 | 0.5803 | 0.3023 | 0.0250 | 0.0000 |
| PARP1 | 0.1820 | 0.5319 | 0.0512 | 0.0000 | 0.2349 |
| STAT5A | 0.2137 | 0.0412 | 0.5421 | 0.2030 | 0.0000 |
| USP39 | 0.0022 | 0.1631 | 0.8231 | 0.0000 | 0.0116 |
| MIOS | 0.0004 | 0.0037 | 0.7192 | 0.2767 | 0.0000 |
| CENPB | 0.2665 | 0.0001 | 0.6384 | 0.0950 | 0.0000 |
| SP1 | 0.0149 | 0.0000 | 0.0002 | 0.9849 | 0.0000 |
| STAT1 | 0.2338 | 0.0000 | 0.0044 | 0.5614 | 0.2004 |
| STAT2 | 0.0000 | 0.0054 | 0.0018 | 0.9928 | 0.0000 |
| MEF2D | 0.0000 | 0.0000 | 0.1295 | 0.8705 | 0.0000 |
| RAB18 | 0.0401 | 0.1691 | 0.0841 | 0.7067 | 0.0001 |
| ABCF2 | 0.0441 | 0.0496 | 0.2173 | 0.6890 | 0.0000 |
| RAN | 0.0076 | 0.0870 | 0.3374 | 0.5514 | 0.0165 |
| IRF1 | 0.0903 | 0.0009 | 0.3781 | 0.5308 | 0.0000 |
| SF1 | 0.2844 | 0.1371 | 0.0194 | 0.5288 | 0.0303 |
| ZBTB1 | 0.0000 | 0.2156 | 0.0054 | 0.2069 | 0.5721 |
| CTNNB1 | 0.0363 | 0.0847 | 0.0557 | 0.0392 | 0.7841 |
| HDAC3 | 0.0997 | 0.1198 | 0.0169 | 0.0563 | 0.7073 |
| GLI4 | 0.0055 | 0.1791 | 0.0767 | 0.0409 | 0.6978 |
| KDM5A | 0.1235 | 0.1629 | 0.1061 | 0.0000 | 0.6075 |
| PSMC2 | 0.2292 | 0.0264 | 0.0778 | 0.1392 | 0.5274 |

**Supplementary Table 3. Lineage-specific regulators of B cells in human fetal hematopoietic differentiation data**

| Genes | Mono vs B cell (DE_logFC) | pDC vs B cell  (DE_logFC) | Mono vs B cell  (DR_logFC) | pDC vs B cell  (DR_logFC) |
| --- | --- | --- | --- | --- |
| HMGA1 | 0.4188 | -0.5327 | 2.4431 | 1.9905 |
| EZR | 1.2987 | 0.6064 | 4.1744 | 9.9767 |
| NFYA | 1.1944 | -0.3260 | 5.6108 | 2.6926 |
| BPTF | 0.5247 | -0.1638 | 7.7830 | 3.1487 |
| NFATC1 | 0.3033 | 0.8311 | 2.2018 | 5.0146 |

DE_logFC refers to the difference in gene expression level compared with the logFC value, and DR_logFC refers to the difference in regulatory weight compared with the logFC value. The above genes only show regulatory differences in the two groups.

**Supplementary Table 4. Lineage-specific regulators of erythroid cells in human fetal hematopoietic differentiation data**

| Genes | Mast vs Ery  (DE_logFC) | Mega vs Ery  (DE_logFC) | Mast vs Ery  (DR_logFC) | Mega vs Ery  (DR_log2FC) |
| --- | --- | --- | --- | --- |
| NUCB1 | -0.5405 | -1.2067 | 4.6820 | 2.6174 |
| MAPK1 | -0.1844 | -0.3408 | 3.0290 | 5.4260 |
| ZNF24 | -0.5101 | -1.0942 | 4.5074 | 1.3179 |
| SF3B1 | -1.1140 | -1.1847 | 3.8172 | 9.4154 |
| KLF13 | 0.3897 | 0.4266 | 2.8528 | 3.0143 |
| FOXO3 | 0.2023 | 1.4999 | 2.2310 | 5.9524 |

DE_logFC refers to the difference in gene expression level compared with the logFC value, and DR_logFC refers to the difference in regulatory weight compared with the logFC value. The above genes only show regulatory differences in the two groups.

**Supplementary Table 5. Lineage-specific regulators of monocytes in human fetal hematopoietic differentiation data**

| Genes | pDC vs Mono  (DE_logFC) | B cell vs Mono  (DE_logFC) | pDC vs Mono  (DR_logFC) | B cell vs Mono  (DR_logFC) |
| --- | --- | --- | --- | --- |
| NFKB1 | 0.0785 | 0.8364 | 1.7322 | 3.6083 |
| ANXA11 | 0.5938 | 0.5994 | 3.0149 | 4.7933 |
| SP1 | -0.0898 | 0.1907 | 2.5424 | 2.7161 |

DE_logFC refers to the difference in gene expression level compared with the logFC value, and DR_logFC refers to the difference in regulatory weight compared with the logFC value. The above genes only show regulatory differences in the two groups.

**Supplementary Table 6. Lineage-specific regulators of pDCs in human fetal hematopoietic differentiation data**

| Genes | Mono vs pDC  (DE_logFC) | B cell vs pDC  (DE_logFC) | Mono vs pDC  (DR_logFC) | B cell vs pDC  (DR_logFC) |
| --- | --- | --- | --- | --- |
| AFF4 | 0.6931 | 0.2446 | 1.2190 | 2.0747 |
| STAT1 | -0.0633 | 0.4865 | 2.3311 | 2.3517 |
| BDP1 | 0.9383 | 0.3991 | 3.1388 | 2.4286 |
| TCF4 | 3.2020 | 0.5695 | 7.8515 | 2.8481 |

DE_logFC refers to the difference in gene expression level compared with the logFC value, and DR_logFC refers to the difference in regulatory weight compared with the logFC value. The above genes only show regulatory differences in the two groups.

**Supplementary Table 7. The prediction performance of CTN under different combinations of GRN inference methods**

|  | hESC | | mESC | |
| --- | --- | --- | --- | --- |
|  | AUROC | AUPRC | AUROC | AUPRC |
| GENIE3 / GENIMS / NARROMI | **0.611** | **0.323** | **0.664** | **0.402** |
| GENIE3 / GENIMS / PPCOR | 0.597 | 0.306 | 0.640 | 0.378 |
| GENIE3 / PPCOR / NARROMI | 0.578 | 0.300 | 0.636 | 0.381 |
| PPCOR / GENIMS / NARROMI | 0.591 | 0.305 | 0.639 | 0.391 |
| GENIE3 | 0.586 | 0.306 | 0.547 | 0.325 |
| GENIMS | 0.540 | 0.276 | 0.494 | 0.298 |
| NARROMI | 0.530 | 0.266 | 0.546 | 0.326 |
| PPCOR | 0.559 | 0.286 | 0.539 | 0.322 |

**Supplementary Table 8. Components of human hematopoietic differentiation data**

| Cell types | Genes | Cells |
| --- | --- | --- |
| Progenitors | 7461 | 3079 |
| Erythroid cells | 7534 | 1842 |
| GMPs | 6947 | 2444 |
| Monocytes | 5471 | 1997 |
| CLPs | 6691 | 864 |

**Supplementary Table 9. Components of human fetal hematopoietic differentiation data**

| Cell types | Genes | Cells |
| --- | --- | --- |
| B cells | 5037 | 735 |
| Erythroid cells | 5040 | 122 |
| GPs | 5040 | 176 |
| Granulocytes | 5040 | 263 |
| HSC-MPPs | 5039 | 1357 |
| LMPs | 5040 | 153 |
| Mast cells | 5040 | 196 |
| Megakaryocytes | 5040 | 96 |
| MEMPs | 5040 | 446 |
| Monocytes | 5038 | 301 |
| pDCs | 5040 | 172 |

**Supplementary Table 10. Components of the normal group and the diseased group in the colorectal cancer data**

| Cell types | Genes | | Cells | |
| --- | --- | --- | --- | --- |
|  | normal | tumor | normal | tumor |
| Epithelial cells | 12443 | 13178 | 1143 | 2060 |
| Stromal cells | 12662 | 13751 | 3560 | 2319 |
| Myeloid cells | 11370 | 11891 | 823 | 926 |
| T cells | 9796 | 10441 | 1535 | 1935 |
| B cells | 10780 | 11195 | 2382 | 690 |

**Supplementary Table 11. Components of the normal group and the diseased group in the lung adenocarcinoma data**

| Cell types | Genes | | Cells | |
| --- | --- | --- | --- | --- |
|  | normal | tumor | normal | tumor |
| B cells | 11041 | 10344 | 613 | 5176 |
| Fibroblasts | 12929 | 13174 | 1650 | 1693 |
| NK cells | 9595 | 10339 | 2548 | 465 |
| T cells | 9562 | 9727 | 3920 | 5124 |

**Supplementary Reference**

[1]	Derecki, N.C., J.C. Cronk, Z. Lu, et al., Wild-type microglia arrest pathology in a mouse model of Rett syndrome*.* Nature, 2012. **484**(7392): 105-9.

[2]	Xu, X., Y. Zhao, Y. Ying, et al., m7G-related genes-NCBP2 and EIF4E3 determine immune contexture in head and neck squamous cell carcinoma by regulating CCL4/CCL5 expression*.* Mol Carcinog, 2023. **62**(8): 1091-1106.

[3]	Chistiakov, D.A., V.A. Myasoedova, V.V. Revin, et al., The impact of interferon-regulatory factors to macrophage differentiation and polarization into M1 and M2*.* Immunobiology, 2018. **223**(1): 101-111.

[4]	Grajkowska, L.T., M. Ceribelli, C.M. Lau, et al., Isoform-Specific Expression and Feedback Regulation of E Protein TCF4 Control Dendritic Cell Lineage Specification*.* Immunity, 2017. **46**(1): 65-77.

[5]	Ippolito, G.C., J.D. Dekker, Y.H. Wang, et al., Dendritic cell fate is determined by BCL11A*.* Proc Natl Acad Sci U S A, 2014. **111**(11): E998-1006.

[6]	Tkachenko, A., K. Kupcova, and O. Havranek, B-Cell Receptor Signaling and Beyond: The Role of Igα (CD79a)/Igβ (CD79b) in Normal and Malignant B Cells*.* Int J Mol Sci, 2023. **25**(1).

[7]	Smillie, C.S., M. Biton, J. Ordovas-Montanes, et al., Intra- and Inter-cellular Rewiring of the Human Colon during Ulcerative Colitis*.* Cell, 2019. **178**(3): 714-730.e22.

[8]	Parkkila, S., P.W. Pan, A. Ward, et al., The calcium-binding protein S100P in normal and malignant human tissues*.* BMC Clin Pathol, 2008. **8**: 2.

[9]	Park, S.Y., J.Y. Kim, G.B. Jang, et al., Aberrant activation of the CD45-Wnt signaling axis promotes stemness and therapy resistance in colorectal cancer cells*.* Theranostics, 2021. **11**(18): 8755-8770.

[10]	Wang, K., Y. Liu, H. Li, et al., Claudin-7 is essential for the maintenance of colonic stem cell homoeostasis via the modulation of Wnt/Notch signalling*.* Cell Death Dis, 2024. **15**(4): 284.

[11]	Facchetti, F., J.K. Chan, W. Zhang, et al., Linker for activation of T cells (LAT), a novel immunohistochemical marker for T cells, NK cells, mast cells, and megakaryocytes: evaluation in normal and pathological conditions*.* Am J Pathol, 1999. **154**(4): 1037-46.

[12]	Yuan, L., J. Xu, Y. Shi, et al., CD3D Is an Independent Prognostic Factor and Correlates With Immune Infiltration in Gastric Cancer*.* Front Oncol, 2022. **12**: 913670.

[13]	Díez-Obrero, V., F. Moratalla-Navarro, G. Ibáñez-Sanz, et al., Transcriptome-Wide Association Study for Inflammatory Bowel Disease Reveals Novel Candidate Susceptibility Genes in Specific Colon Subsites and Tissue Categories*.* J Crohns Colitis, 2022. **16**(2): 275-285.

[14]	Tuxhorn, J.A., S.J. McAlhany, T.D. Dang, et al., Stromal cells promote angiogenesis and growth of human prostate tumors in a differential reactive stroma (DRS) xenograft model*.* Cancer Res, 2002. **62**(11): 3298-307.

[15]	Kikutani, H., A. Yokota, N. Uchibayashi, et al., Structure and function of Fc epsilon receptor II (Fc epsilon RII/CD23): a point of contact between the effector phase of allergy and B cell differentiation*.* Ciba Found Symp, 1989. **147**: 23-31; discussion 31-5.

[16]	Harrison, O.J. and F.M. Powrie, Regulatory T cells and immune tolerance in the intestine*.* Cold Spring Harb Perspect Biol, 2013. **5**(7).

[17]	Bhattacharyya, A., R. Chattopadhyay, S. Mitra, et al., Oxidative stress: an essential factor in the pathogenesis of gastrointestinal mucosal diseases*.* Physiol Rev, 2014. **94**(2): 329-54.

[18]	Packiriswamy, N., K.F. Coulson, S.J. Holcombe, et al., Oxidative stress-induced mitochondrial dysfunction in a normal colon epithelial cell line*.* World J Gastroenterol, 2017. **23**(19): 3427-3439.

[19]	Zhang, H.S., Y. Chen, L. Fan, et al., The Endoplasmic Reticulum Stress Sensor IRE1α in Intestinal Epithelial Cells Is Essential for Protecting against Colitis*.* J Biol Chem, 2015. **290**(24): 15327-36.

[20]	Wang, H. and J.J. Unternaehrer, Epithelial-mesenchymal Transition and Cancer Stem Cells: At the Crossroads of Differentiation and Dedifferentiation*.* Dev Dyn, 2019. **248**(1): 10-20.

[21]	Shan, G., W. Wang, X. Li, et al., Epigenetic-targeted biomimetic nanomedicine modulates epithelial mesenchymal transition to enhance chemosensitivity in heterogeneous tumors*.* Biomaterials, 2025. **324**: 123529.

[22]	Ezine, E., C. Lebbe, and N. Dumaz, Unmasking the tumourigenic role of SIN1/MAPKAP1 in the mTOR complex 2*.* Clin Transl Med, 2023. **13**(10): e1464.

[23]	Kim, N., H.K. Kim, K. Lee, et al., Single-cell RNA sequencing demonstrates the molecular and cellular reprogramming of metastatic lung adenocarcinoma*.* Nat Commun, 2020. **11**(1): 2285.

[24]	Greenberg, Z.J., D.A. Monlish, R.L. Bartnett, et al., The Tetraspanin CD53 Regulates Early B Cell Development by Promoting IL-7R Signaling*.* J Immunol, 2020. **204**(1): 58-67.

[25]	Chen, Y., C. Chen, J. Shih, et al., P2.04-34 FCGR2B Expression as a Regulator of Immunity in Non-Small Cell Lung Cancer Patients*.* Journal of Thoracic Oncology, 2019. **14**(10): S721-S722.

[26]	Lankry, D., H. Simic, Y. Klieger, et al., Expression and Function of CD300 in NK Cells*.* The Journal of Immunology, 2010. **185**(5): 2877-2886.

[27]	Dong, C., Z. Lin, Y. Hu, et al., KLRD1 (CD94): A Prognostic Biomarker and Therapeutic Candidate in Head and Neck Squamous Cell Carcinoma*.* J Cancer, 2025. **16**(3): 982-995.

[28]	Laletin, V., P.-L. Bernard, C. Montersino, et al., DOK1 and DOK2 regulate CD8 T cell signaling and memory formation without affecting tumor cell killing*.* Scientific Reports, 2024. **14**(1): 15053.

[29]	Kato, A., K.E. Hulse, B.K. Tan, et al., B-lymphocyte lineage cells and the respiratory system*.* J Allergy Clin Immunol, 2013. **131**(4): 933-57; quiz 958.

[30]	Guidi, R., D. Xu, D.F. Choy, et al., Steroid-induced fibroblast growth factors drive an epithelial-mesenchymal inflammatory axis in severe asthma*.* Sci Transl Med, 2022. **14**(641): eabl8146.

[31]	Culley, F.J., Natural killer cells in infection and inflammation of the lung*.* Immunology, 2009. **128**(2): 151-63.

[32]	Dong, C., Cytokine Regulation and Function in T Cells*.* Annu Rev Immunol, 2021. **39**: 51-76.

[33]	Herrera-Quiterio, G.A., H.A. Valencia-González, K.G. de la Cruz-López, et al., TMEM160 Promotes Tumor Growth in Lung Adenocarcinoma and Cervical Adenocarcinoma Cell Lines*.* Int J Mol Sci, 2025. **26**(3).

[34]	Lin, T.Y., C.H. Yang, H.C. Chou, et al., EGFR Mutation-Harboring Lung Cancer Cells Produce CLEC11A with Endothelial Trophic and Tumor-Promoting Activities*.* Cancers (Basel), 2022. **14**(5).

[35]	Kim, S., ppcor: An R Package for a Fast Calculation to Semi-partial Correlation Coefficients*.* Commun Stat Appl Methods, 2015. **22**(6): 665-674.

[36]	Chu, L.F., N. Leng, J. Zhang, et al., Single-cell RNA-seq reveals novel regulators of human embryonic stem cell differentiation to definitive endoderm*.* Genome Biol, 2016. **17**(1): 173.

[37]	Camp, J.G., K. Sekine, T. Gerber, et al., Multilineage communication regulates human liver bud development from pluripotency*.* Nature, 2017. **546**(7659): 533-538.

[38]	Weng, Y., S. Han, M.T. Sekyi, et al., Self-Assembled Matrigel-Free iPSC-Derived Liver Organoids Demonstrate Wide-Ranging Highly Differentiated Liver Functions*.* Stem Cells, 2023. **41**(2): 126-139.

[39]	Hayashi, T., H. Ozaki, Y. Sasagawa, et al., Single-cell full-length total RNA sequencing uncovers dynamics of recursive splicing and enhancer RNAs*.* Nat Commun, 2018. **9**(1): 619.

[40]	Loh, C.H., S. van Genesen, M. Perino, et al., Loss of PRC2 subunits primes lineage choice during exit of pluripotency*.* Nat Commun, 2021. **12**(1): 6985.
